# Prehospital optimal shock energy for defibrillation (POSED): A cluster randomised controlled feasibility trial

**DOI:** 10.1016/j.resplu.2024.100569

**Published:** 2024-02-09

**Authors:** Helen Pocock, Charles D Deakin, Ranjit Lall, Felix Michelet, Chu Sun, Deb Smith, Catherine Hill, Jeskaran Rai, Kath Starr, Martina Brown, Isabel Rodriguez-Bachiller, Gavin D. Perkins

**Affiliations:** a South Central Ambulance NHS Foundation Trust, Talisman Way, Bicester, Oxfordshire OX26 6HR, UK; b Warwick Clinical Trials Unit, University of Warwick, Gibbet Hill Road, Coventry, CV4 7AL, Warwickshire, UK; c University Hospitals Southampton NHS Foundation Trust, Tremona Road, Southampton, Hampshire, UK; d University Hospitals Birmingham NHS Foundation Trust, Mindelsohn Way, Edgbaston, Birmingham B15 2GW, Warwickshire, UK

**Keywords:** Defibrillation, Out-of-hospital Cardiac Arrest, Ventricular Fibrillation, Electric Countershock, Cardiopulmonary Resuscitation, Feasibility study

## Abstract

**Background:**

We explored the feasibility of a large-scale UK ambulance services trial of optimal defibrillation shock energy for out-of-hospital cardiac arrest. The primary objective of this feasibility study was to establish the number of eligible patients and the number recruited. Secondary outcomes were adherence to allocated treatment and data completeness.

**Methods:**

We conducted a three-arm parallel group cluster randomised controlled feasibility study in a single ambulance service in southern England. Adult patients in out-of-hospital cardiac arrest treated for a shockable rhythm were included. Zoll X series defibrillators (clusters) were randomised to deliver 120–150–200 J, 150–200–200 J, or 200–200–200 J shock strategies.

**Results:**

Between March 2022 and February 2023, we randomised 38 eligible patients (120–150–200 J (n = 12), 150–200–200 J (n = 10), 200–200–200 J (n = 16)) to the study. The recruitment rate per cluster was 0.07 per month. The median patient age was 71 years (IQR 59–81 years); 79% were male. Twenty-eight cardiac arrests (74%) occurred in a private residence, 29 (76%) were witnessed and 32 (84%) patients received bystander CPR. Treatment adherence was 93% and completeness of clinical and electrical outcomes was 86%. At 30 days, 3/36 (8.3%) patients survived; we were unable to collect survival outcomes for two patients. Defibrillation data collection became difficult when defibrillators became separated from their allocated vehicles.

**Conclusion:**

We have demonstrated the feasibility of a cluster randomised controlled trial of optimal shock energy for defibrillation in a UK ambulance service. We have identified possible solutions to issues relating to trial design.

## Background

Globally, it is estimated that between 6.5% and 37.8% of patients in out-of-hospital cardiac arrest (OHCA) present in a shockable rhythm on first assessment,^1^ and a further 8% of those initially in a non-shockable rhythm may convert to a shockable rhythm during the resuscitation. ^2^ For these patients, defibrillation is an essential precursor to restoration of circulation and forms an essential link in the chain of survival. ^3^ There is a fundamental need to identify optimal shock energy for the earliest shocks. Within the first 6 shocks there is an exponential decline in the chance of achieving sustained spontaneous circulation.^4^ The chance of survival to 30 days decreases by 10% with each successive shock, and most survival benefit is derived within the first three shocks.^5^ However, there is currently no clear evidence for an optimal energy strategy.^6^

In the absence of clear evidence, European Resuscitation Council (ERC) guidelines indicate that any energy level within the range 120–360 J is acceptable for the initial shock, with a fixed or escalating strategy for subsequent shocks.^7^, ^8^ The International Liaison Committee on Resuscitation (ILCOR) has identified defibrillation energy levels as a priority area for research.^8^ Here we report a feasibility study for a pragmatic clinical effectiveness randomised controlled trial of optimal shock strategy.

The Prehospital Optimal Shock Energy for Defibrillation (POSED) study aimed to explore the feasibility of a multi-centre randomised controlled trial to identify the optimal shock strategy for OHCA. Our primary objective was to establish the number of eligible patients and the number recruited. Secondary objectives were to measure the rate of treatment adherence and to identify the best outcome measures by reviewing data completeness.

## Methods

### Trial design

We conducted a three-arm parallel group cluster randomised controlled feasibility study in a single UK NHS Ambulance Service between 22nd March 2022 and 28th February 2023. The unit of randomisation was a defibrillator. Defibrillators were programmed to deliver one of three shock strategies: 120–150–200 J, 150–200–200 J or 200–200–200 J. During the study we refined the definition of a cluster to *a defibrillator carried by its associated ambulance vehicle* since it was only possible to track vehicles and not defibrillators and we found that, on occasion, defibrillators became separated from their associated vehicles. Other changes are described in the Table S1 in the Supplementary materials.

The study was approved by the London (Harrow) NHS Research Ethics Committee (20/LO/1242) and prospectively registered on the ISRCTN Trial Registry (ISRCTN16327029). It was sponsored by the University of Warwick. A summary of the study protocol is published in a peer-reviewed journal.^9^ The full protocol is available in the supplementary material. We conducted the study in accordance with ICH Good Clinical Practice guidelines, the UK Policy Framework for Health and Social Care Research,^10^ and University of Warwick Standard Operating Procedures. We report according to CONSORT guidelines for feasibility trials cluster randomised trials.^11^, ^12^

### Patient and public involvement

Six patient and public partners were involved throughout the project, bringing experience as a relative of a cardiac arrest survivor, recipient of NHS cardiac care, previous training in the use of defibrillators, first response to 999 callers, and prior experience of representing patients and public views.

### Eligibility criteria

Patients were eligible to be included if they sustained an OHCA attended by a crew from South Central Ambulance Service NHS Foundation Trust, resuscitation was attempted and a shock delivered at any time during the resuscitation attempt. Patients known or suspected to be under 18 years old were excluded. Enrolled patients were identified through electronic reports generated by the electronic patient record (ePR) system. All cases where *cardiac arrest* was recorded on the ePR, either in ‘presenting complaint’ or ‘impression’ fields, or where shocks or cardiac arrest medications were administered were screened by the ambulance service research team for eligibility. During set-up, information about the trial was delivered to ambulance staff providing the study intervention. In accordance with the UK Mental Capacity Act, patients were enrolled without prior consent; deferred consent for continuation in the study was sought after the initial emergency had passed and patients were on a ward.

### Setting

The study was conducted in the north region of South Central Ambulance Service (Berkshire, Oxfordshire and Buckinghamshire). The whole service covers a population of around 7 million. The service answers 1.2 million emergency calls and attends 4890 patients sustaining an out-of-hospital cardiac arrest each year. Depending on proximity to the incident, the ambulance service first response consisted of a single-crewed response car, double-crewed ambulance or a single-crewed team leader vehicle (or may be second tier response providing team management).

### Interventions

All patients received standard resuscitation treatment according to Resuscitation Council UK guidelines and Joint Royal Colleges Ambulance Liaison Committee guidelines.^6^, ^13^ The exception was that defibrillation energies were randomly allocated. Zoll X series defibrillators were pre-programmed to deliver one of the randomised shock energy strategies. Consequently, ambulance staff did not have to deviate from their routine practice when delivering patient treatment. However, manual override (and therefore treatment non-adherence) remained possible.

When a shock was required during treatment, ambulance staff manually charged defibrillators. The pre-programmed shock energy was applied whether in manual or AED mode and small impedance-compensating adjustments made automatically by the device as usual.

### Outcomes

The primary outcome was the number of eligible patients and the number recruited. Secondary outcomes included rate of adherence to the allocated treatment and data completeness of clinical and process outcomes. These are further described in the Supplementary data, Table S2.

### Randomisation

Randomisation was stratified by vehicle type (ambulance, response car and team leader vehicle) in a 1:1:1 ratio. The random allocation was computer generated, and the randomisation list prepared by the study statistician who ensured that allocation could not be predicted by vehicle type.

This was an open-label study as ambulance staff would see the energy level on charging the defibrillator. Since the defibrillators were pre-programmed, assignment of the intervention depended on which device was first available at the patient’s side. Ambulance control room staff, hospital staff and patients were blind to the treatment allocation. Programming of defibrillators and outcome assessment were performed by the same researcher (HP) but outcomes checked by a blinded assessor (CD).

### Sample size & statistical analysis

We aimed to recruit patients for a period of two years or until 90 patients were reached, ideally 30 in each arm (i.e., a recruitment rate of 3.75 patients per month or 0.1 per month per cluster). This would align with a recommended sample size of at least 50 for feasibility studies, ^14^ whilst allowing roughly equal recruitment into each arm. We estimated that to meet this target we would need to include ambulance vehicles from a single geographical node, thereby recruiting 36 clusters.

Descriptive statistics are presented, as the study is not powered to detect a difference in treatment arms. For all outcomes, categorical data are presented as frequency and percentage and continuous data as median and interquartile range. Data are analysed on an intention to treat basis.

## Results

### Cluster recruitment

Although we aimed to randomise 36 clusters, during the conduct of the study a further 13 clusters were randomised to offset clusters unavailable due to servicing and repair, taking the total to 49.

### Patient recruitment

The start of the trial was delayed by 13 months due to the COVID-19 pandemic. Funding constraints precluded trial extension, therefore the recruitment period was shorter than anticipated (11 months recruitment compared to the planned 24 months).

**Fig. 1:**
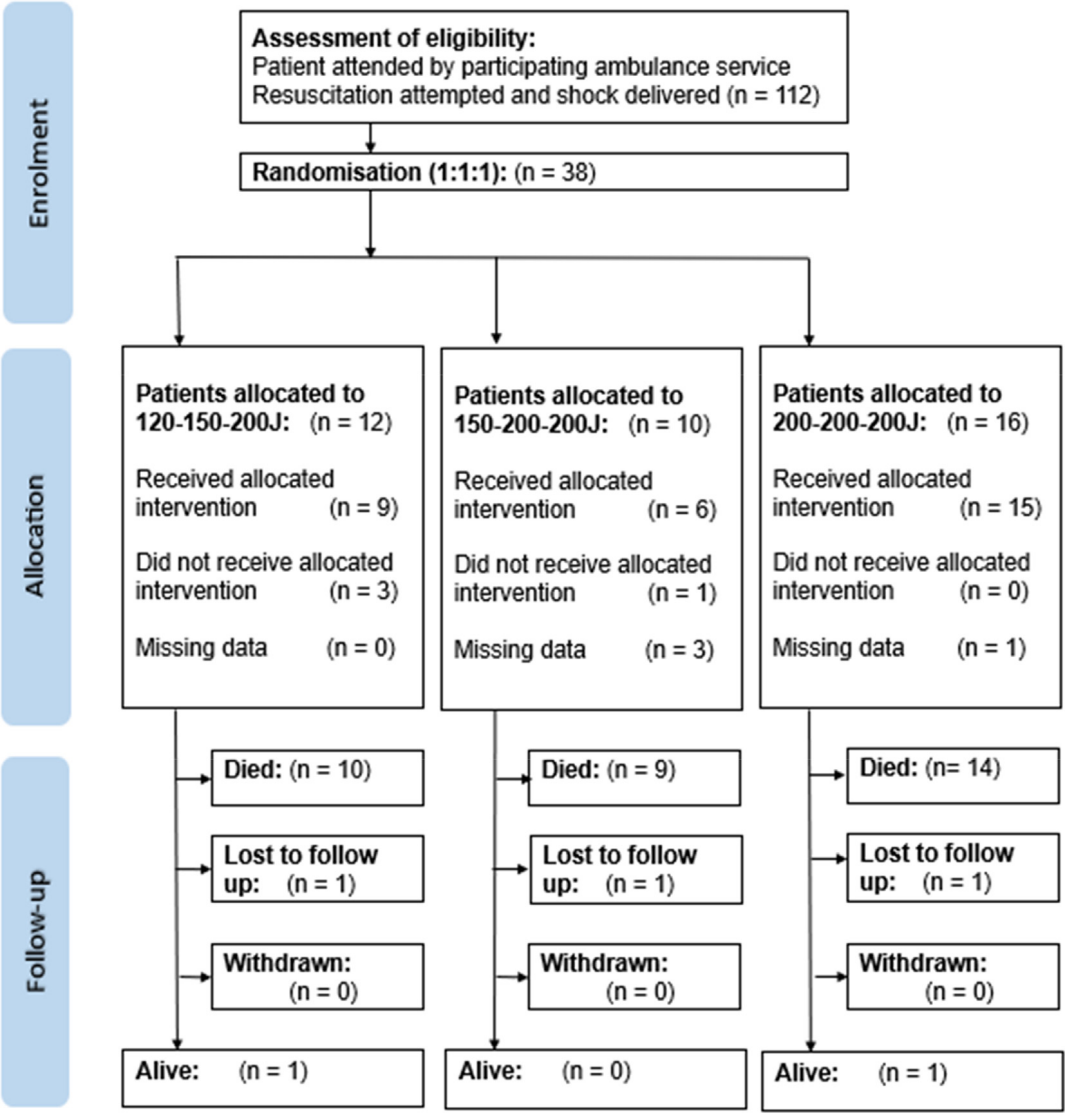
CONSORT diagram.

We identified 112 potentially eligible patients with OHCA attended by ambulances initially allocated to carry randomised defibrillators. Patient flow is shown in Fig. 1 and shows reasons for exclusion. Of the 38 enrolled patients, 12 received 120–150–200 J strategy, 10 received 150–200–200 J strategy and 16 received 200–200–200 J strategy.

### Patient baseline demographics

We were able to collect all baseline data apart from one case where we were unable to ascertain the time of first shock. Table 1 shows the sample baseline demographics.

**Table 1:** Patient baseline characteristics.

**Variable**	**All cases (n = 38)**	**Data completeness**
Age (median [IQR])	71 (59–81)	100%
Male	30 (79%)	100%
Location of arrest		100%
Private residence	28 (74%)	
Public place	10 (26%)	
Other	0 (0%)	
Witnessed event	29 (76%)	100%
EMS	3 (8%)	
Bystander	26 (68%)	
Unwitnessed	9 (24%)	
Bystander CPR*	32 (84%)	100%
Prior shocks delivered by non-study defibrillator	4 (11%)	100%
Initial rhythm		100%
VF/pVT	25 (66%)	
Asystole	8 (21%)	
PEA	5 (13%)	
Time from call to application of defibrillator (median [IQR])	11.6 (8.1–14.4 min)	97.4%
Presumed cardiac aetiology	37 (97%)	100%

IQR = Interquartile range; EMS = Emergency Medical System staff; VF = ventricular fibrillation; pVT = pulseless Ventricular Tachycardia.

* Bystander CPR = Non-EMS witnessed events receiving bystander CPR.

### Primary outcome

**Fig. 2:**
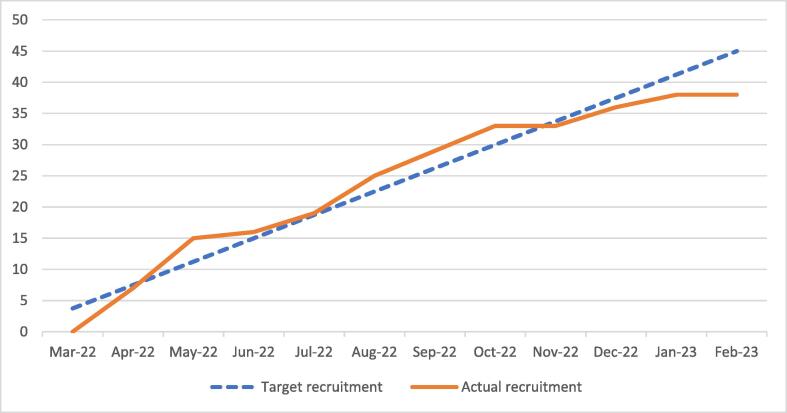
Recruitment rate. Recruitment was restricted to 11 months due to COVID-19.

Of the 112 eligible patients, 38 received a randomised intervention (34%). Over the 11 months the trial recruited, the observed recruitment rate per cluster (defibrillator) was 0.07 patients per month and we recruited 3.5 patients per month compared to a target of 0.1 patients per cluster / 3.75 patients per month. Fig. 2 shows the cumulative recruitment over the 11 month period.

During the conduct of the trial, 46 of the 49 defibrillators were unavailable at various times. We identified 43 occasions when the associated vehicle was unavailable due to servicing or repair and 18 occasions where a defibrillator had been moved to another vehicle. We were only able to track cases by vehicle callsign and not by defibrillator and we came across instances where we identified potential enrolments but when the researcher went to retrieve data, they found that vehicle carried a non-study defibrillator. Since the shock had been delivered by a non-randomised defibrillator the patient had not been recruited into the study. We noted the cases where this occurred. Defibrillator movement / unavailability led to the loss of 74 patients (6.7 patients per month (0.14 per cluster per month). The potential recruitment rate for the 49 defibrillators was therefore 0.21 patients (0.07 + 0.14) per cluster per month.

Figure S1 in the Supplementary materials shows the number of randomised clusters (defibrillators carried on their associated vehicles) that were available to the trial by month (i.e., excluding those that had gone for repair, been moved to the wrong vehicle, and those housed on vehicles that were off the road for service or repair). The recruitment rate per cluster for ambulances was 0.08 per month, for rapid response cars 0.05 and for team leader vehicles 0.02 per month.

Table 2 summarises the observed and potential recruitment rates per defibrillator. We calculate that if all defibrillators (n = 160) were randomised within an ambulance service trust, and no eligible patients were lost due to movements between vehicles, then the recruitment rate across South Central Ambulance Service would be 33.6 per month (based on 0.21 per cluster per month).

**Table 2:** Primary outcome - Observed (and projected) recruitment rates per cluster.

	**Monthly recruitment rate per cluster**	**Annual recruitment rate per cluster**
**Observed**	0.07	0.71
**Potential (had there been no defibrillator/vehicle separation)**	0.21	2.52

### Secondary outcomes

#### Adherence rate

For the secondary outcome of treatment adherence, of the 81 shocks where the administered energy was known, 75/81 (93%) were delivered at the allocated treatment energy. First shock energies were known in 34 cases and were 26/34 (77%) adherent. Second shocks were received by 25 patients and third shocks by 22 and their combined adherence was 44/47 (94%).

#### Data completeness clinical outcomes

Table 3 shows the ability to collect both clinical and electrical outcome data. For the secondary outcome of data completeness of clinical outcomes, overall data completeness was 85.9% (range 81.8–100%), with 86.2% of electrical and 84.6% of clinical outcomes having been collected.

**Table 3:** Secondary outcomes - Patient Outcomes.

	Pooled event rate*	**Data completeness**
1st shock ROOR (2 min post shock)	15.6%	32/38 (84%)
2nd shock ROOR (2 min post shock)^a^	12%	25/29 (86%)
3rd shock ROOR (2 min post shock)^b^	10%	20/26 (77%)
1st shock resulting rhythm (2 min post shock):		33/38 (87%)
VF	52.6%	
pVT	10.5%	
PEA	7.9%	
Asystole	7.9%	
2nd shock resulting rhythm (2 min post shock)^a^		25/29 (86%)
VF	62.1%	
pVT	10.3%	
PEA	13.8%	
Asystole	0%	
3rd shock resulting rhythm (2 min post shock)^b^		21/26 (81%)
VF	57.7%	
pVT	19.2%	
PEA	3.8%	
Asystole	0%	
Re-arrest rate (refibrillation during the out-of-hospital phase)	60.5%	37/38 (97%)
Survived event (ROSC at hospital handover)	28.9%	14/14 (100%)
Survived to discharge	13.2%	14/14 (100%)
Survived to 30 days^c^	8.3%	12/14 (86%)
Survival with favourable neurological outcome (mRS ≥ 3) at discharge	5.3%	2/5 (40%)
Survival with favourable neurological outcome (mRS ≥ 3) at 30 days	2.6%	2/5 (40%)

ROOR = Return of Organised Rhythm; VF = Ventricular Fibrillation; pVT = Pulseless Ventricular Tachycardia; PEA = Pulseless Electrical Activity; ROSC = Return of Spontaneous Circulation; mRS = Modified Rankin Scale score.

* Number of cases where this was observed combining all treatment arms.

a Calculated with N = No. of patients who received ≥ 2 shocks = 29.

b Calculated with N = No. of patients who received ≥ 3 shocks = 26.

c The survival status (alive) of one patient who declined follow up was carried forward from day 21 to 30 days.

We were unable to collect ROOR data in 16 cases (6 following first, 4 following second and 6 following third shocks). In one case we could not establish presence or absence of refibrillation due to missing defibrillator data. We collected survival data up to hospital discharge in 100% of cases. We were unable to meet with three patients in hospital to seek consent for follow up and so sent invitation letters. One declined follow-up and two did not respond. Data presented by treatment arm can be found in Tables S3-S6 in the Supplementary data.

#### Data completeness for process outcomes

Data completeness ranged from 65.8% to 100% for the process outcomes (Table 4). The main reasons for missing data were either that it was not recorded by the defibrillator because an accelerometer pad was not used (these were being gradually introduced into the ambulance service during the study), or that the full defibrillator data record had been overwritten. Most parameters were within guideline-recommended limits apart from chest compression depth which was too shallow.

**Table 4:** Secondary outcomes - Process outcomes.

	**Pooled event rate**	**Data completeness**
Quality of CPR, *median (IQR):*		
Chest compression rate (cpm)	105.8 (102.1–114.0)	25/38 (65.8%)
Chest compression depth (cm)	4.3 (3.8 – 5.3)	25/38 (65.8%)
Chest compression fraction (%)	88.0 (79.8–90.6)	25/38 (65.8%)
Pre-shock pause (s)	2.0 (1.0–4.0)	77/93^a^(82.8%)
Post-shock pause (s)	2.0 (1.0–3.0)	77/93^a^ (82.8%)
Total number of pre-hospital shocks delivered:	4 (2–6)	38/38 (100%)
Advanced airway applied, no. (%)		36/38 (94.8%)
Supraglottic airway or endotracheal tube, no. (%)	10 (26.3%)	
Supraglottic airway only, no. (%)	22 (57.9%)	
Endotracheal tube only, no. (%)	1 (2.6%)	
Intravenous medications administered, no. (%)		38/38 (100%)
Adrenaline and amiodarone, no. (%)	19 (50.0)	
Adrenaline only, no. (%)	17 (44.7)	
Amiodarone only, no. (%)	0 (0.0)	
Transported to hospital (%)	36.8%	38/38 (100%)

IQR = Interquartile range; cpm = compressions per minute; s = seconds.

a N = 9*1 + 3*2 + 26*3 = 93 (9 patients received 1 shock only, 3 patients receive 2 shocks in total, 26 patients received >=3 shocks. We only collect the data within 3 shocks).

Shock data and other treatment data were more reliably recorded. All 38 patients received at least one shock. If the shock energy could not be verified via patient clinical record, defibrillator data download or printout, data were deemed missing. Overall, only 6/81 (7%) of shock energy data were missing.

#### Harms

No adverse events were reported and of the four protocol deviations that occurred, two patients received shock energy strategy from a different treatment arm from that allocated and two received incorrect shock strategy (although energies were 120/150/200 J).

Sub-group analyses were not undertaken since there were insufficient data.

### Patients and public

The patient and public (PPI) advisory panel felt that the public would support a trial. They felt that the feasibility study had been extremely valuable in identifying problems and suggesting solutions, especially in recognising the value of a potential remote data download solution for defibrillators.

## Discussion

The key finding from this trial was that implementation of the POSED protocol was feasible in a single NHS Ambulance Service. Although this was a cluster randomised trial, we could not compute a cluster effect due to the small sample size. The observed recruitment rate was 3.5 patients per month (0.07 per cluster (defibrillator) per month). Modelling identified this would increase to 33.6 patients per month (0.21 per cluster per month) if all defibrillators in the ambulance service were randomised and available. Treatment adherence and data completeness overall were high at 93% and 85.9% respectively. Sample size calculations were modelled on change in effect size (Odds Ratio) of 1.2 or 1.25 based on previous defibrillation studies, 15, 16 or minimal clinically important difference (MCID) of 5% based on clinician recommendations.^17^ These indicated that a definitive trial would require between 691 and 15,762 patients (see S7-S9 in the Supplementary data). Based on the survival rate we observed (13%), and allowing for a 5% loss to follow up, a total sample size of 3288 would be required for a 3-armed trial with 90% power, to detect a 5% change in outcome from a baseline of 13% to 18%. Figure S2 in the Supplementary data shows projected recruitment for a full trial assuming extrapolated to all 11 ambulance services, allowing for a staggered set-up phase (one site opening per month). Assuming each ambulance service randomised the same number of clusters (160), this would take the 11 Ambulance services of England and Wales 14 months to achieve. The study oversight committee and patient and public partners engaged throughout the study were supportive of progressing to a definitive trial.

The population and outcomes are broadly generalisable to other out-of-hospital research settings where studies compared outcomes of shocks whether or not the initial rhythm was shockable. In POSED, 18% of patients with OHCA received shocks, which is comparable with incidence in the Out-of-hospital Rectilinear Biphasic Trial (ORBIT) and the DEFI 2005 Randomised controlled trial of the effect of automated external defibrillator CPR protocol on outcome from OHCA (DEFI 2005) studies (18% and 24% respectively).^15^, ^16^ Outcomes for POSED compare favourably to the ORBIT study (13.2% vs. 6.7% for survival to hospital discharge, 8.3% vs. 6.2% for survival to 30 days and 5.7% vs. 2.9% for favourable neurological outcome at discharge).^16^ But survival to discharge (8.3%) was lower than that reported in the DEFI study (12%).^15^ For the outcome survival to discharge we found relatively small effect sizes with wide confidence intervals (OR 150–200–200 J = 0.56 [95% CI, 0.043 to 7.21]; OR 200–200–200 J = 0.71 [95% CI, 0.086 to 5.959]; comparator in each case 120–150–200 J). This lack of precision is due to the small sample size.

This cluster-randomised study used the defibrillator as the unit of randomisation, in common with other defibrillation studies.^18^, ^19^ Other studies have randomised by station,^15^, ^16^ although this would not have worked in this study due to the large degree of movement of vehicles and defibrillators between stations. One prior study achieved individual randomisation by returning the defibrillator to a supervisor for reprogramming after each use.^20^ Although a preferable model of randomisation, this would not have been feasible in POSED due to operational demand rendering each vehicle and its allocated defibrillator in near constant use. Treatment adherence (93%) was higher than other cluster-randomised out-of-hospital resuscitation studies, such as PARAMEDIC (85%)^21^, TIMBER (88%)^20^, and ORBIT (80%)^16^. Reasons for non-adherence tended to be similar across the studies: lack of availability of device or switching to another device during treatment. The former of these was most frequently encountered in POSED. The slightly higher adherence rates (95%) seen in the Optimised Response to Cardiac Arrest (ORCA) study,^18^ may have been due to the system of daily allocation of tagged devices, a practice that could not be accommodated in the high-demand current study setting.

A strength of our study is that it enables consideration of progression to a full trial to take account not only of metrics such as recruitment rate, but also of inherent practicalities and challenges. The key challenge was defibrillators becoming separated from their allocated vehicles, making tracking of defibrillator use virtually impossible. Ensuring the success of this top-down approach to case identification and data collection would require either ensuring that defibrillators and vehicles remain together, or immediate transfer of data to a storage system that may be interrogated retrospectively. Alternatively, a bottom-up approach, whereby ambulance crews notify researchers of an enrolment, device serial number and its current location, would support the timely identification of enrolled patients. In a main trial, randomising all defibrillators would negate the need for tracking as long as data download was not delayed. Crew notification would minimise this delay. This would be further supported by upload to a data storage cloud. Unfortunately, at the time of the study, cloud based-technology was incompatible with the EMS IT systems. Now that the system is more mature this limitation could be overcome.

This feasibility study was subject to several limitations. Firstly, we were unable to achieve our planned sample size. This was largely due to pandemic-related delays but means that our point estimates are less precise than intended. Secondly, we do not know how representative our single site was of other UK ambulance services or those outside of the UK. Other sites may be less or more able to implement the study protocol. Thirdly, our follow-up rate was lower than anticipated. This may have been improved by identifying all patients within a shorter timeframe and by allowing for a repeat invitation letter to be sent to patients.

## Conclusion

We have demonstrated the feasibility of a pragmatic cluster randomised controlled trial of prehospital optimal shock energy for defibrillation in a UK ambulance service. We have recognised that timely identification of study enrolments and robust data collection methods are essential to optimise delivery of the study protocol.

## CRediT authorship contribution statement

**Helen Pocock:** Writing – original draft, Visualization, Project administration, Methodology, Investigation, Funding acquisition, Conceptualization. **Charles D Deakin:** Writing – review & editing, Writing – original draft, Supervision, Methodology, Funding acquisition, Conceptualization. **Ranjit Lall:** Writing – review & editing, Writing – original draft, Supervision, Methodology, Funding acquisition, Formal analysis. **Felix Michelet:** Writing – review & editing, Methodology. **Chu Sun:** Writing – review & editing, Visualization, Methodology. **Deb Smith:** Writing – review & editing, Methodology. **Catherine Hill:** Writing – review & editing, Project administration, Methodology. **Jeskaran Rai:** Writing – review & editing, Project administration, Methodology. **Kath Starr:** Writing – review & editing, Project administration, Methodology. **Martina Brown:** Writing – review & editing, Project administration. **Isabel Rodriguez-Bachiller:** Writing – review & editing, Investigation. **Gavin D. Perkins:** Writing – review & editing, Writing – original draft, Supervision, Methodology, Funding acquisition, Conceptualization.

## Declaration of competing interest

The authors declare the following financial interests/personal relationships which may be considered as potential competing interests: HP is a member of the International Liaison Committee on Resuscitation (ILCOR) Advanced Life Support task force committee. She holds a grant from the National Institute for Health and Social Care Research (NIHR)/Health Education England. GDP is Editor for Resuscitation and Resuscitation Plus and holds grants from the NIHR, British Heart Foundation, Resuscitation Council UK (RCUK) and Laerdal. He is an employee of University of Warwick, University Hospital of Coventry and Warwick, University Hospital Birmingham and West Midlands Ambulance Service and holds Leadership roles with ILCOR, the European Resuscitation Council, and RCUK. CDD, RL, DS, CS, FM, CH, JR, KS, MB, IR: Nil.
