# Supplementary material for: Prehospital optimal shock energy for defibrillation (POSED): A cluster randomised controlled feasibility trial
Source: Resusc Plus. 2024 Feb 9;17:100569. doi: 10.1016/j.resplu.2024.100569 (PMC10869912; doi:10.1016/j.resplu.2024.100569)
Supplement: Supplementary data 1 [file mmc1.docx]

**Supplementary Data**

**Figure S1: Number of available clusters and recruitment rate per cluster each month**


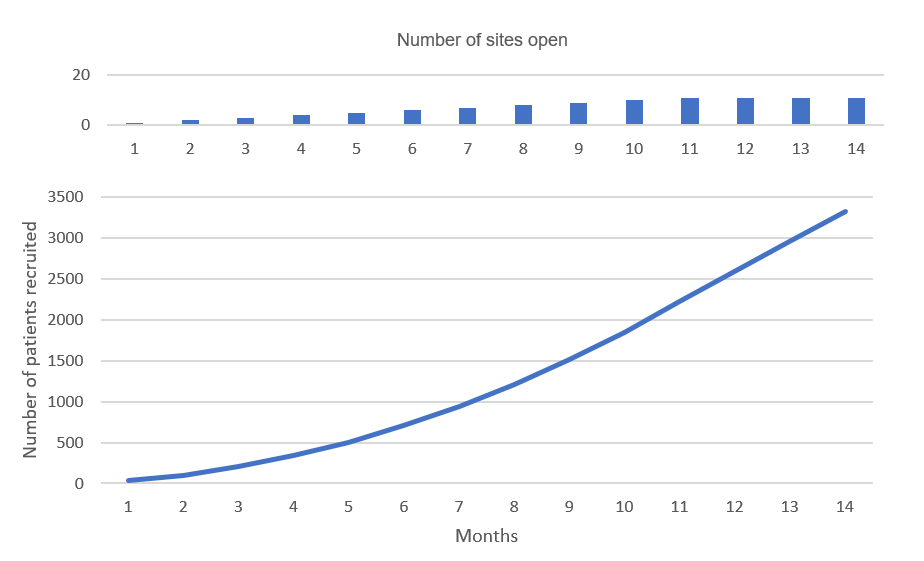


**Figure S2:** **Projected recruitment rate based on 11 Ambulance Services (sites)**

**Table S1: Differences between registry record, protocol and study report**

|  | **Source, date of change and explanation** | | |
| --- | --- | --- | --- |
| **Item** | **Trial registration record** | **Health Research Authority approved protocol (v5.0_12 Dec 2022)** | **Study report** |
| Intervention | **Original:**  *“This study will involve putting defibrillators into one of three groups to deliver one of the three different shock strategies in current UK use. All other treatments and care will be given in the usual way. The three groups will treat the same number of patients (30 each). To try to make sure the groups are the same to start with, each defibrillator will be put into a group by chance (randomly).  Whether patients receive the shock strategy requested, what other care the patient received and whether the patients survive to 30 days will be collected for the three groups. It is possible to collect this data without the patient having to do anything, as long as the patient does not object to us collecting this data from their medical notes. By looking at ambulance service 999 call records the number of patients that could have been included will be compared to the actual number included to find the ‘recruitment rate’.  All patients who survive the cardiac arrest will be contacted by the Research Paramedic in hospital once they are on the ward and the initial emergency has passed. The Research Paramedic will explain the trial and that the patient has been included. The Research Paramedic will give the patient an information sheet to read. When the patient has had time to read the sheet the Research Paramedic will answer any questions and check with the patient if they are happy for us to continue to collect data and take part in the follow up. If someone does not have the mental capacity to consent, the Research Paramedic will inform their next of kin, known as the personal consultee, and ask if they think that the patient would be happy to take part.  All surviving patients will be invited to take part in the follow up which happens 30 days after the cardiac arrest. This will involve a research paramedic scoring how well they have recovered either by talking with the patient or by reading their medical notes.  I will also speak to the ambulance staff involved to find out what makes it easier or more difficult to record what happens to patients after treatment. Where I find difficulties, I will try to work out how I can reduce these difficulties so that a large study will work.  Members of the public have helped us to decide how we should let patients and their relatives know that they have been included in the study. Sadly, most of the patients in the study will not survive. We were not sure how to let the relatives of these patients know that they were included in the study. Members of the public told us that we should write a letter. They have helped to design these letters so that they are easy for patients and relatives to understand.”*  **Revised: 11/07/2022**  *“This study will involve putting defibrillators into one of three groups to deliver one of the three different shock strategies in current UK use. All other treatments and care will be given in the usual way. The three groups will treat the same number of patients (30 each). To try to make sure the groups are the same to start with, each defibrillator will be put into a group by chance (randomly).  Whether patients receive the shock strategy requested, what other care the patient received and whether the patients survive to hospital discharge and to 30 days will be collected for the three groups. It is possible to collect this data without the patient having to do anything, as long as the patient does not object to us collecting this data from their medical notes. By looking at ambulance service 999 call records the number of patients that could have been included will be compared to the actual number included to find the ‘recruitment rate’.  All patients who survive the cardiac arrest will be contacted by the Research Paramedic in hospital once they are on the ward and the initial emergency has passed. The Research Paramedic will explain the trial and that the patient has been included. The Research Paramedic will give the patient an information sheet to read. When the patient has had time to read the sheet the Research Paramedic will answer any questions and check with the patient if they are happy for us to continue to collect data and take part in the follow up. If someone does not have the mental capacity to consent, the Research Paramedic will inform their next of kin, known as the personal consultee, and ask if they think that the patient would be happy to take part.  All surviving patients will be invited to take part in the follow up which happens at hospital discharge and at 30 days after the cardiac arrest. This will involve a research paramedic scoring how well they have recovered either by talking with the patient or by reading their medical notes.  I will also speak to the ambulance staff involved to find out what makes it easier or more difficult to record what happens to patients after treatment. Where I find difficulties, I will try to work out how I can reduce these difficulties so that a large study will work.  Members of the public have helped us to decide how we should let patients and their relatives know that they have been included in the study. Sadly, most of the patients in the study will not survive. We were not sure how to let the relatives of these patients know that they were included in the study. Members of the public told us that we should write a letter. They have helped to design these letters so that they are easy for patients and relatives to understand.”*  **Rationale:** Following approval for a non-substantial protocol amendment made for consistency so that each patient would be assessed at the same time points, i.e. both at discharge and at 30 days.  **Revised: 08/01/2024**  *“This study will involve putting defibrillators into one of three groups to deliver one of the three different shock strategies in current UK use. All other treatments and care will be given in the usual way. The three groups will treat the same number of patients (30 each). To try to make sure the groups are the same to start with, each defibrillator will be put into a group by chance (randomly).  Whether patients receive the shock strategy requested, what other care the patient received and whether the patients survive to hospital discharge and to 30 days will be collected for the three groups. It is possible to collect this data without the patient having to do anything, as long as the patient does not object to us collecting this data from their medical notes. By looking at ambulance service 999 call records the number of patients that could have been included will be compared to the actual number included to find the ‘recruitment rate’.  All patients who survive the cardiac arrest will be contacted by the Research Paramedic in hospital once they are on the ward and the initial emergency has passed. The Research Paramedic will explain the trial and that the patient has been included. The Research Paramedic will give the patient an information sheet to read. When the patient has had time to read the sheet the Research Paramedic will answer any questions and check with the patient if they are happy for us to continue to collect data and take part in the follow up. If someone does not have the mental capacity to consent, the Research Paramedic will inform their next of kin, known as the personal consultee, and ask if they think that the patient would be happy to take part.*  *All surviving patients will be invited to take part in the follow up which happens at hospital discharge and at 30 days after the cardiac arrest. This will involve a research paramedic scoring how well they have recovered either by talking with the patient or by reading their medical notes.*  *Members of the public have helped us to decide how we should let patients and their relatives know that they have been included in the study. Sadly, most of the patients in the study will not survive. We were not sure how to let the relatives of these patients know that they were included in the study. Members of the public told us that we should write a letter. They have helped to design these letters so that they are easy for patients and relatives to understand.”*  **Rationale:**  Removal of nested qualitative sub-study. Due to the COVID-19 pandemic it was not possible to complete this work. | Consistent with registry record apart from the addition of a nested qualitative study, section 3.4, aiming to explore and describe the experience of paramedics delivering this study, specifically the barriers and facilitators to patient recruitment.  **Rationale:**  Due to the COVID-19 pandemic it was not possible to complete this work. | Consistent with registry record. |
| Primary outcome measure | **Original:**  *“Recruitment rate recorded as the number of eligible participants enrolled in the study by 24 months”*  **Revised:** **21/07/2023**  *“Recruitment rate calculated from the number of eligible participants and the number recruited in the study by 24 months”*  **Rationale:**  Typographical error: change from ‘participants’ to ‘patients’.  Removal of timeframe as due to the COVID-19 pandemic it was not possible to run the study for 24 months.  **Revised: 08/01/2024**  *“The number of eligible patients and the number recruited”*  **Rationale:**  Alignment with approved study protocol.  Removal of timeframe as due to the COVID-19 pandemic it was not possible to run the study for 24 months. | Primary feasibility outcome  • No. of eligible patients and no. of patients recruited | The primary outcome was the number of eligible patients and the number recruited.  Consistent with registry record. |
| Secondary outcome measures | **Original: *“****1. Treatment adherence rate recorded as the proportion of patients receiving the allocated treatment prior to hospital admission 2. Data completeness of clinical outcomes, defined as the proportion of enrolled patients who by the end of trial have data relating to: 2.1. Neurological outcomes (mRS score) measured using the Rankin Focused Assessment at 30 days post cardiac arrest 2.2. Return Of Organised Rhythm capable of sustaining a pulse (ROOR) assessed using defibrillator data recorded 2 min post shock 2.3. Resulting rhythm (VF/pVT/PEA/asystole) assessed using defibrillator data recorded 2 min post shock 2.4. Re-arrest rate (re-fibrillation) assessed using defibrillator data recorded during the out-of-hospital phase of resuscitation 2.5. Survival assessed using hospital records at 30 days post cardiac arrest 3. Data completeness of process outcomes: 3.1. Quality of CPR (chest compression rate, chest compression depth, chest compression fraction, pre-shock pause, post-shock pause) measured using defibrillator data from the out-of-hospital phase of care 3.2. Number of shocks measured using defibrillator data from the out-of-hospital phase of care 3.3. Advanced airway applied (% advanced airway applied and % supraglottic airway or endotracheal tube) measured using patient clinical records from the out-of-hospital phase of care 3.4. Intravenous medicines administered (% cases where medicines administered and % adrenaline, amiodarone) measured using patient clinical records from the out-of-hospital phase of care 3.5. Transported to hospital (% transported) measured using Ambulance Service data from the out-of-hospital phase of care”*  **Revision 1:** **21/07/2023**  *“1. Treatment adherence rate recorded as the proportion of patients receiving the allocated treatment prior to hospital admission 2. Data completeness of clinical outcomes, defined as the proportion of enrolled patients who by the end of trial have data relating to: 2.1. Return Of Organised Rhythm capable of sustaining a pulse (ROOR) assessed using defibrillator data recorded 2 min post-shock 2.2. Resulting rhythm (VF/pVT/PEA/asystole) assessed using defibrillator data recorded 2 min post-shock 2.3. Re-arrest rate (re-fibrillation) assessed using defibrillator data recorded during the out-of-hospital phase of resuscitation 2.4. Survived event (return of spontaneous circulation (ROSC)) using ambulance records at hospital handover 2.5. Survival assessed using hospital records at hospital discharge and at 30 days post cardiac arrest 2.6 Neurological outcomes (mRS score) measured using the Rankin Focused Assessment at hospital discharge and 30 days post cardiac arrest”*  **Rationale:** Order of outcomes amended for consistency with protocol.  **Revision 2:** **31/07/2023**  *“1. Treatment adherence rate recorded as the proportion of patients receiving the allocated treatment prior to hospital admission 2. Data completeness of clinical outcomes, defined as the proportion of enrolled patients who by the end of trial have data relating to: 2.1. Return Of Organised Rhythm capable of sustaining a pulse (ROOR) assessed using defibrillator data recorded 2 min post-shock 2.2. Resulting rhythm (VF/pVT/PEA/asystole) assessed using defibrillator data recorded 2 min post-shock 2.3. Re-arrest rate (re-fibrillation) assessed using defibrillator data recorded during the out-of-hospital phase of resuscitation 2.4. Survived event (return of spontaneous circulation (ROSC)) using ambulance records at hospital handover 2.5. Survival assessed using hospital records at hospital discharge and at 30 days post cardiac arrest 2.6 Neurological outcomes (mRS score) measured using the Rankin Focused Assessment at hospital discharge and 30 days post cardiac arrest 3. Data completeness of process outcomes: 3.1. Quality of CPR (chest compression rate, chest compression depth, chest compression fraction, pre-shock pause, post-shock pause) measured using defibrillator data from the out-of-hospital phase of care 3.2. Number of shocks measured using defibrillator data from the out-of-hospital phase of care 3.3. Advanced airway applied (% advanced airway applied and % supraglottic airway or endotracheal tube) measured using patient clinical records from the out-of-hospital phase of care 3.4. Intravenous medicines administered (% cases where medicines administered and % adrenaline, amiodarone) measured using patient clinical records from the out-of-hospital phase of care 3.5. Transported to hospital (% transported) measured using Ambulance Service data from the out-of-hospital phase of care”*  **Rationale:** Process outcomes included as secondary outcomes for consistency with protocol. | Secondary feasibility outcomes  • Data completeness of clinical outcomes below  • Treatment adherence rate  • Acceptability of approach to informing relatives of non-survivors  • Issues identified by ambulance staff and suggestions for study optimisation.  **Differences:**  • Acceptability of approach to informing relatives of non-survivors.  • Issues identified by ambulance staff and suggestions for study optimisation.  **Rationale:**  Our assessment of the acceptability of approach to informing relatives will be reported elsewhere.  Due to the COVID-19 pandemic it was not possible to investigate issues identified by ambulance staff and suggestions for study optimisation. | *“Secondary outcomes included rate of adherence to the allocated treatment and data completeness of clinical and process outcomes”.*    Consistent with registry record. |
| Participant inclusion criteria | **Original:** *“1. Patients suffering OHCA attended by a crew from participating ambulance service 2. Resuscitation attempted and shock indicated as per Resuscitation Council (UK) and JRCALC guidelines”*  **Revised: 21/07/2023**  *“1. Patients suffering OHCA attended by a crew from participating ambulance service 2. Resuscitation attempted and shock delivered as per Resuscitation Council (UK) and JRCALC guidelines”*  **Rationale:** Indicated vs. delivered changed in *POSED protocol v2.0_15Apr2021*) | Consistent with registry record. | “Patients were eligible to be included if they sustained an OHCA attended by a crew from South Central Ambulance Service NHS Foundation Trust, resuscitation was attempted and a shock delivered at any time during the resuscitation attempt.”  **Difference:**  Participating ambulance service named. |
| Recruitment start date | **Original:**  *“01/07/2021”*  **Multiple revisions. Final revision: 25/03/2022**  *“28/03/2022”*  Rationale: The COVID-19 pandemic resulted in a number of changes to study design. This required protocol amendment and further ethical review and delayed the opening of the study. The delays meant that another study was due to open at the same time | N/A | Consistent with registry record. |
| Recruitment end date | **Original:**  *“30/06/2023”*  **Revised: 05/04/2023**  *“28/02/2023”*  **Rationale:**  Projected end date corrected in response to clarification of funding extension due to delays in opening study. | N/A | Consistent with registry record. |
| Study end date | **Original:**  *“30/12/2023”*  **Revised: 31/07/2023**  *“28/02/2023”*  **Rationale:**  End date amended in line with recruitment end date. | N/A | Consistent with registry record. |

**Table S2: Secondary outcomes**

| **Outcome** | **Description** |
| --- | --- |
| Treatment adherence | Treatment was considered adherent when the delivered shock energy was the same as that allocated. The adherence of individual shocks was considered rather than combined shocks per patient. |
| Clinical outcomes:   1. Electrical efficacy | - Return of organised rhythm (ROOR) - Resulting rhythm two minutes after each shock - Refibrillation at any point in the out-of-hospital phase.   These were assessed from defibrillator data downloads. Defibrillators could store only 150 data records before being overwritten. Treatment summary printouts were an alternative source of shock delivery timings. |
| 1. Clinical effectiveness | - Survival to hospital handover - Survival to hospital discharge - Survival to 30 days post-arrest   These were assessed via ambulance, hospital or General Practitioner (GP) patient clinical records or through patient assessment.   - Neurological outcome at hospital discharge - Neurological outcome at 30 days   These were obtained through research paramedic or GP assessment of the patient using the Rankin Focussed Assessment*. |
| Process outcomes | Quality of CPR:   - Chest compression rate - Chest compression depth - Chest compression fraction - Pre-shock pause - Post-shock pause - Total number of prehospital shocks   These were assessed from defibrillator data downloads. The ePR was an alternative source of total number of shocks.  Other resuscitation interventions:   - Advanced airway applied - Medications administered   These were assessed from ambulance patient clinical records (ePR).  Conveyance to hospital.  This was ascertained from ambulance patient clinical records (ePR). |

*ROOR = Return of Organised Rhythm; ePR = electronic Patient Record.*

**Rankin Focused Assessment = a standardised tool reducing inter-rater variation of the modified Rankin Scale which scores global disability from 0 (no symptoms) to 6 (dead). Scores of 0 – 3 are usually classified as good and 4 – 6 as poor. Citation: Saver JL, Bogdan F, Hamilton S, et al. Improving the reliability of stroke disability grading in clinical trials and clinical practice: the Rankin Focused Assessment (RFA. Stroke; 41(5): 992-995. doi: 10.1161/STROKEAHA.109.571364.*

**Table S3: Survival outcomes at hospital discharge and 30 days**

| **Variable** | **120-150-200J**  **(n = 12)** | **150-200-200J**  **(n = 10)** | **200-200-200J**  **(n = 16)** | **All Cases**  **(N = 38)** |
| --- | --- | --- | --- | --- |
| **At hospital handover**, No. (%) | | | | |
| ROSC | 4 (33.3) | 2 (20.0) | 5 (31.2) | 11 (28.9) |
| CPR ongoing | 1 (8.3) | 0 (0.0) | 2 (12.5) | 3 (7.9) |
| Not conveyed | 7 (58.3) | 8 (80.0) | 9 (56.2) | 24 (63.2) |
| Missing | 0 (0.0) | 0 (0.0) | 0 (0.0) | 0 (0.0) |
| **At discharge**, No. (%) | | | | |
| Alive | 2 (16.7) | 1 (10.0) | 2 (12.5) | 5 (13.2) |
| Died in hospital | 3 (25.0) | 1 (10.0) | 5 (31.2) | 9 (23.7) |
| Died pre-hospital | 7 (58.3) | 8 (80.0) | 9 (56.2) | 24 (63.2) |
| Missing | 0 (0.0) | 0 (0.0) | 0 (0.0) | 0 (0.0) |
| **At 30 days**, No. (%) | | | | |
| Alive | 1 (8.3) | 0 (0.0) | 1 (6.2) | 3 (7.9) |
| Died | 10 (83.3) | 9 (90.0) | 14 (87.5) | 33 (86.8) |
| Missing | 1 (8.3) | 1 (10.0) | 1 (6.2) | 2 (5.3) |

*ROSC = Return of Spontaneous Circulation; CPR = Cardiopulmonary Resuscitation*

**Table S4: Neurological outcome at hospital discharge and 30 days**

| **Variable** | **120-150-200J**  **(n = 12)** | **150-200-200J**  **(n = 10)** | **200-200-200J**  **(n = 16)** | **All Cases**  **(N = 38)** |
| --- | --- | --- | --- | --- |
| **mRS score at discharge**, No. (%) | | | | |
| 0 | 0 (0.0) | 0 (0.0) | 0 (0.0) | 0 (0.0) |
| 1 | 0 (0.0) | 0 (0.0) | 0 (0.0) | 0 (0.0) |
| 2 | 0 (0.0) | 0 (0.0) | 1 (6.2) | 1 (2.6) |
| 3 | 1 (8.3) | 0 (0.0) | 0 (0.0) | 1 (2.6) |
| 4 | 0 (0.0) | 0 (0.0) | 0 (0.0) | 0 (0.0) |
| 5 | 0 (0.0) | 0 (0.0) | 0 (0.0) | 0 (0.0) |
| 6 (died) | 10 (83.3) | 9 (90.0) | 14 (87.5) | 33 (86.8) |
| Missing | 1 (8.3) | 1 (10.0) | 1 (6.2) | 3 (7.9) |
| **mRS score at 30 days**, No. (%) | | | | |
| 0 | 0 (0.0) | 0 (0.0) | 0 (0.0) | 0 (0.0) |
| 1 | 0 (0.0) | 0 (0.0) | 0 (0.0) | 0 (0.0) |
| 2 | 1 (8.3) | 0 (0.0) | 0 (0.0) | 1 (2.6) |
| 3 | 0 (0.0) | 0 (0.0) | 0 (0.0) | 0 (0.0) |
| 4 | 0 (0.0) | 0 (0.0) | 1 (6.2) | 1 (2.6) |
| 5 | 0 (0.0) | 0 (0.0) | 0 (0.0) | 0 (0.0) |
| 6 (died) | 10 (83.3) | 9 (90.0) | 14 (87.5) | 33 (86.8) |
| Missing | 1 (8.3) | 1 (10.0) | 1 (6.2) | 3 (7.9) |

*mRS = modified Rankin Scale.*

**Table S5: Post-shock rhythms (2 minutes post-shock)**

| **Variable** | **120-150-200J**  **(n = 12)** | **150-200-200J**  **(n = 10)** | **200-200-200J**  **(n = 16)** | **All Cases**  **(N = 38)** |
| --- | --- | --- | --- | --- |
| **Rhythm post 1st shock**, No. (%) | | | | |
| ROSC | 1 (8.3) | 0 (0.0) | 2 (12.5) | 3 (7.9) |
| pVT | 1 (8.3) | 1 (10.0) | 2 (12.5) | 4 (10.5) |
| VF | 7 (58.3) | 6 (60.0) | 7 (43.8) | 20 (52.6) |
| PEA | 1 (8.3) | 0 (0.0) | 2 (12.5) | 3 (7.9) |
| Asystole | 2 (16.7) | 0 (0.0) | 1 (6.2) | 3 (7.9) |
| Missing | 0 (0.0) | 3 (30.0) | 2 (12.5) | 5 (13.2) |
| **Rhythm post 2nd shock**, No. (%) ^a^ | | | | |
|  | (n=10) | (n=9) | (n=10) | (n=29) |
| ROSC | 0 (0.0) | 0 (0.0) | 0 (0.0) | 0 (0.0) |
| pVT | 1 (10.0) | 0 (0.0) | 2 (20.0) | 3 (10.3) |
| VF | 6 (60.0) | 4 (44.4) | 8 (80.0) | 18 (62.1) |
| PEA | 3 (30.0) | 1 (11.1) | 0 (0.0) | 4 (13.8) |
| Asystole | 0 (0.0) | 0 (0.0) | 0 (0.0) | 0 (0.0) |
| Missing | 0 (0.0) | 4 (44.4) | 0 (0.0) | 4 (13.8) |
| **Rhythm post 3rd shock**, No. (%) ^b^ | | | | |
|  | (n=9) | (n=8) | (n=9) | (n=26) |
| ROSC | 0 (0.0) | 0 (0.0) | 0 (0.0) | 0 (0.0) |
| pVT | 1 (11.1) | 0 (0.0) | 4 (44.4) | 5 (19.2) |
| VF | 7 (77.8) | 4 (50.0) | 4 (44.4) | 15 (57.7) |
| PEA | 1 (11.1) | 0 (0.0) | 0 (0.0) | 1 (3.8) |
| Asystole | 0 (0.0) | 0 (0.0) | 0 (0.0) | 0 (0.0) |
| Missing | 0 (0.0) | 4 (50.0) | 1 (11.1) | 5 (19.2) |

*ROSC = Return of Spontaneous Circulation; pVT = Pulseless Ventricular Tachycardia; VF = Ventricular Fibrillation; PEA = Pulseless Electrical Activity;*

**Table S6: Treatment adherence by shock**

| **Variable** | **120-150-200J**  **(n = 12)** | **150-200-200J**  **(n = 10)** | **200-200-200J**  **(n = 16)** | **All Cases**  **(N = 38)** |
| --- | --- | --- | --- | --- |
| **1st shock**, No. (%) | | | | |
| Delivery of allocated energy | 9 (75.0) | 6 (60.0) | 15 (93.8) | 30 (78.9) |
| Non-adherence | 3 (25.0) | 1 (10.0) | 0 (0.0) | 4 (10.5) |
| Not given | 0 (0.0) | 0 (0.0) | 0 (0.0) | 0 (0.0) |
| Missing | 0 (0.0) | 3 (30.0) | 1 (6.2) | 4 (10.5) |
| **2nd shock**, No. (%) | | | | |
| Delivery of allocated energy | 9 (75.0) | 5 (50.0) | 10 (62.5) | 24 (63.2) |
| Non-adherence | 1 (8.3) | 0 (0.0) | 0 (0.0) | 1 (2.6) |
| Not given | 2 (16.7) ^a^ | 2 (20.0) ^b^ | 6 (37.5) ^c^ | 10 (26.3) |
| Missing | 0 (0.0) | 3 (30.0) | 0 (0.0) | 3 (7.9) |
| **3rd shock**, No. (%) | | | | |
| Delivery of allocated energy | 8 (66.7) | 5 (50.0) | 8 (50.0) | 21 (55.3) |
| Non-adherence | 1 (8.3) | 0 (0.0) | 0 (0.0) | 1 (2.6) |
| Not given | 3 (25.0) ^d^ | 2 (20.0) ^e^ | 8 (50.0) ^f^ | 13 (34.2) |
| Missing | 0 (0.0) | 3 (30.0) | 0 (0.0) | 3 (7.9) |

*^a^ Group A: 2 patients received 1 shock only.*

*^b^ Group B: 1 patient received 1 shock only. 1 patient received 2 shocks in total but only one of these was from SCAS.*

*^c^ Group C: 6 patients received 1 shock only.*

*^d^ Group A: 3 patients received 1 or 2 shocks only.*

*^e^ Group B: 2 patients received 1 or 2 shocks only.*

*^f^ Group C: 7 patients received 1 or 2 shocks only. 1 patient received 4 shocks in total but only two of these were from SCAS.*

**S7: Sample size calculation based on effect size change for survival to discharge/ binary neurological outcome**

The odds ratio for survival to discharge for patients receiving 150-200-200J versus 120-150-200J strategy was 0.56 (95% CI, 0.043 to 7.21). The odds ratio for patients receiving 200-200-200J versus 120-150-200J was 0.71 (95% CI, 0.086 to 5.959). Both comparisons show a small effect size with wide confidence intervals, indicating a lack of precision, due to the small sample size. To estimate a sample size with greater precision we refer to two other defibrillation studies using the same outcome. In the DEFI study, the odds ratio for survival to discharge for the intervention was 1.25 (95% CI, 0.828 to 1.898.(15) In the ORBIT study the odds ratio was 1.2 (95% CI, 0.408 to 3.547).(16)

**Table S7a: Sample sizes for a binary outcome based on change in outcome detected in ORBIT (Morrison, 2005)**

| **p1** | **p2** | **OR** | **Total sample size for 2-armed trial** | | **Total sample size for 3-armed trial** | |
| --- | --- | --- | --- | --- | --- | --- |
|  |  |  | **80% power** | **90% power** | **80% power** | **90% power** |
| 13% | 15.2% | 1.2 | 7850 | 10508 | 11775 | 15762 |
| 14% | 16.3% | 1.2 | 7372 | 9868 | 11058 | 14802 |
| 15% | 17.5% | 1.2 | 6936 | 9286 | 10404 | 13929 |
| 16% | 18.6% | 1.2 | 6638 | 8886 | 9957 | 13329 |
| 17% | 19.7% | 1.2 | 6308 | 8446 | 9462 | 12669 |
| 18% | 20.9% | 1.2 | 6042 | 8090 | 9063 | 12135 |
| 19% | 22.0% | 1.2 | 5790 | 7752 | 8685 | 11628 |
| 20% | 23.1% | 1.2 | 5586 | 7478 | 8379 | 11217 |

*p1 = baseline proportion of sample with outcome of interest (i.e. in a sample not receiving the intervention); p2 = proportion of sample with outcome of interest in sample receiving the intervention; OR = Odds Ratio.*

**Table S7b: Sample sizes for a binary outcome based on change in outcome detected in DEFI (Jost, 2010)**

| **p1** | **p2** | **OR** | **Total sample size for 2-armed trial** | | **Total sample size for 3-armed trial** | |
| --- | --- | --- | --- | --- | --- | --- |
|  |  |  | **80% power** | **90% power** | **80% power** | **90% power** |
| 13% | 15.7% | 1.25 | 5138 | 6880 | 7707 | 10320 |
| 14% | 16.9% | 1.25 | 4838 | 6476 | 7257 | 9714 |
| 15% | 18.1% | 1.25 | 4590 | 6144 | 6885 | 9216 |
| 16% | 19.2% | 1.25 | 4360 | 5836 | 6540 | 8754 |
| 17% | 20.4% | 1.25 | 4170 | 5582 | 6255 | 8373 |
| 18% | 21.5% | 1.25 | 3988 | 5340 | 5982 | 8010 |
| 19% | 22.7% | 1.25 | 3838 | 5138 | 5757 | 7707 |
| 20% | 23.8% | 1.25 | 3694 | 4944 | 5541 | 7416 |

*p1 = baseline proportion of sample with outcome of interest (i.e. in a sample not receiving the intervention); p2 = proportion of sample with outcome of interest in sample receiving the intervention; OR = Odds Ratio.*

**S8: Sample size calculation based on effect size for ordinal neurological outcome**

There were an insufficient number of patients in whom mRS score had been assessed in POSED to inform a sample size calculation. Instead, we took the baseline outcome from the PARAMEDIC trial, where survival with good neurological outcome in the control group was 6%.(21) PARAMEDIC used Cerebral Performance Category (CPC) score as the neurological outcome which is a five-point scale, whereas mRS is a seven-point scale so this works as an approximation but is a limitation of this surrogate approach. Table S6 shows an estimate of sample size modelled on PARAMEDIC trial data. Loss to follow up was extremely low in PARAMEDIC (1 out of 2819 in the control arm) and so this has not been adjusted for in these calculations.

**Table S8: Sample sizes for an ordinal outcome based on change in outcome in PARAMEDIC (Perkins, 2015)**

| **p1** | **p2** | **OR** | **Total sample size** | |
| --- | --- | --- | --- | --- |
|  |  |  | **80% power** | **90% power** |
| 6% | 7.20% | 1.21 | 13309 | 17817 |
| 6% | 7.30% | 1.23 | 11162 | 14943 |
| 6% | 7.40% | 1.25 | 9440 | 12637 |
| 6% | 7.60% | 1.29 | 8098 | 10841 |
| 6% | 7.70% | 1.31 | 7032 | 9414 |
| 6% | 7.80% | 1.33 | 6170 | 8260 |

*p1 = baseline proportion of sample with outcome of interest (i.e. in a sample not receiving the intervention); p2 = proportion of sample with outcome of interest in sample receiving the intervention; OR = Odds Ratio.*

**S9: Sample size calculations based on minimal clinically important difference in survival to discharge or binary neurological outcome**

Emergency physicians and acute cardiovascular researchers identified that a change of 5% in survival or favourable neurological outcome was the minimal clinically important difference required to change practice.(17)

**Table S9a: Sample size for a binary outcome considering Minimal Clinically Important Difference**

| **p1** | **p2** | **One arm** | | **Total Size (3 arms)** | |
| --- | --- | --- | --- | --- | --- |
|  |  | **80% power** | **90% power** | **80% power** | **90% power** |
| 5% | 10% | 432 | 578 | 1296 | 1734 |
| 6% | 11% | 485 | 649 | 1455 | 1947 |
| 7% | 12% | 536 | 718 | 1608 | 2154 |
| 10% | 15% | 683 | 915 | 2049 | 2745 |
| 13% | 18% | 819 | 1096 | 2457 | 3288 |

*p1 = baseline proportion of sample with outcome of interest (i.e. in a sample not receiving the intervention); p2 = proportion of sample with outcome of interest in sample receiving the intervention;*

**Table 9b: Sample size for an ordinal outcome considering** **Minimal Clinically Important Difference**

| **p1** | **p2** | **Total sample size (3 arms)** | |
| --- | --- | --- | --- |
|  |  | **80% power** | **90% power** |
| 5% | 10% | 691 | 925 |
| 6% | 11% | 925 | 1238 |

*p1 = baseline proportion of sample with outcome of interest*

*(i.e. in a sample not receiving the intervention); p2 = proportion*

*of sample with outcome of interest in sample receiving the*

*intervention;*

**S10: CONSORT checklist**

| Section/Topic | Item No | Checklist item | Reported on page No |
| --- | --- | --- | --- |
| Title and abstract | | | |
|  | 1a | Identification as a pilot or feasibility randomised trial in the title | Title page |
|  | 1b | Structured summary of pilot trial design, methods, results, and conclusions (for specific guidance see CONSORT abstract extension for pilot trials) | Abstract, p.1 |
| Introduction | | | |
| Background and objectives | 2a | Scientific background and explanation of rationale for future definitive trial, and reasons for randomised pilot trial | Background, p.2 |
|  | 2b | Specific objectives or research questions for pilot trial | Background, p.2 |
| Methods | | | |
| Trial design | 3a | Description of pilot trial design (such as parallel, factorial) including allocation ratio | Methods (*Trial design*), p.2 |
|  | 3b | Important changes to methods after pilot trial commencement (such as eligibility criteria), with reasons | Methods (*Trial design*), p.2 |
| Participants | 4a | Eligibility criteria for participants | Methods (*Eligibility criteria*), p.3 |
|  | 4b | Settings and locations where the data were collected | Methods (*Eligibility criteria*), p.3 |
|  | 4c | How participants were identified and consented | Methods (*Eligibility criteria*), p.3; Study protocol, referred to in Methods (*Trial design*), p.3 |
| Interventions | 5 | The interventions for each group with sufficient details to allow replication, including how and when they were actually administered | Methods (*Trial design*), p.2; Methods (*Interventions*), p.3 |
| Outcomes | 6a | Completely defined prespecified assessments or measurements to address each pilot trial objective specified in 2b, including how and when they were assessed | Methods (*Outcomes*), p.3-4 |
|  | 6b | Any changes to pilot trial assessments or measurements after the pilot trial commenced, with reasons | N/A |
|  | 6c | If applicable, prespecified criteria used to judge whether, or how, to proceed with future definitive trial | N/A |
| Sample size | 7a | Rationale for numbers in the pilot trial | Methods (*Sample size & statistical analysis*), p.4 |
|  | 7b | When applicable, explanation of any interim analyses and stopping guidelines | N/A |
| Randomisation: |  |  |  |
| Sequence  generation | 8a | Method used to generate the random allocation sequence | Methods (*Randomisation*), p.4 |
|  | 8b | Type of randomisation(s); details of any restriction (such as blocking and block size) | Methods (*Randomisation*), p.4 |
| Allocation  concealment  mechanism | 9 | Mechanism used to implement the random allocation sequence (such as sequentially numbered containers), describing any steps taken to conceal the sequence until interventions were assigned | Methods (*Randomisation*), p.4 |
| Implementation | 10 | Who generated the random allocation sequence, who enrolled participants, and who assigned participants to interventions | Methods (*Randomisation*), p.4 |
| Blinding | 11a | If done, who was blinded after assignment to interventions (for example, participants, care providers, those assessing outcomes) and how | Methods (*Randomisation*), p.4 |
|  | 11b | If relevant, description of the similarity of interventions | N/A |
| Statistical methods | 12 | Methods used to address each pilot trial objective whether qualitative or quantitative | Methods (*Outcomes*), p.3 |
| Results | | | |
| Participant flow (a diagram is strongly recommended) | 13a | For each group, the numbers of participants who were approached and/or assessed for eligibility, randomly assigned, received intended treatment, and were assessed for each objective | Figure 1 |
|  | 13b | For each group, losses and exclusions after randomisation, together with reasons | Figure 1 |
| Recruitment | 14a | Dates defining the periods of recruitment and follow-up | Results (*Patient recruitment*), p.5 |
|  | 14b | Why the pilot trial ended or was stopped | Results (*Patient recruitment*), p.5 |
| Baseline data | 15 | A table showing baseline demographic and clinical characteristics for each group | Table 1 |
| Numbers analysed | 16 | For each objective, number of participants (denominator) included in each analysis. If relevant, these numbers should be by randomised group | Results (*Secondary outcomes*), p.10 |
| Outcomes and estimation | 17 | For each objective, results including expressions of uncertainty (such as 95% confidence interval) for any  estimates. If relevant, these results should be by randomised group | Table 4; Discussion, p.15 |
| Ancillary analyses | 18 | Results of any other analyses performed that could be used to inform the future definitive trial | Discussion, p.14, Figure 4 |
| Harms | 19 | All important harms or unintended effects in each group (for specific guidance see CONSORT for harms) | Results (*Secondary outcomes (Harms)*), p.14 |
|  | 19a | If relevant, other important unintended consequences | N/A |
| Discussion | | | |
| Limitations | 20 | Pilot trial limitations, addressing sources of potential bias and remaining uncertainty about feasibility | Discussion, p.16 |
| Generalisability | 21 | Generalisability (applicability) of pilot trial methods and findings to future definitive trial and other studies | Discussion, p.16 |
| Interpretation | 22 | Interpretation consistent with pilot trial objectives and findings, balancing potential benefits and harms, and considering other relevant evidence | Discussion, p.16 |
|  | 22a | Implications for progression from pilot to future definitive trial, including any proposed amendments | Discussion, p.16 |
| Other information | | |  |
| Registration | 23 | Registration number for pilot trial and name of trial registry | Methods (*Trial design*), p.3 |
| Protocol | 24 | Where the pilot trial protocol can be accessed, if available | Methods (*Trial design*), p.3 |
| Funding | 25 | Sources of funding and other support (such as supply of drugs), role of funders | Funding, p.17 |
|  | 26 | Ethical approval or approval by research review committee, confirmed with reference number | Methods (*Trial design*), p.3 |

Citation: Eldridge SM, Chan CL, Campbell MJ, Bond CM, Hopewell S, Thabane L, et al. CONSORT 2010 statement: extension to randomised pilot and feasibility trials. BMJ. 2016;355. This is an Open Access article distributed in accordance with the terms of the Creative Commons Attribution (CC BY 3.0) license (<http://creativecommons.org/licenses/by/3.0/>), which permits others to distribute, remix, adapt and build upon this work, for commercial use, provided the original work is properly cited.
