# Supplementary material for: Prehospital optimal shock energy for defibrillation (POSED): A cluster randomised controlled feasibility trial
Source: Resusc Plus. 2024 Feb 9;17:100569. doi: 10.1016/j.resplu.2024.100569 (PMC10869912; doi:10.1016/j.resplu.2024.100569)
Supplement: Supplementary data 2 [file mmc2.pdf]

POSED
Prehospital Optimal Shock Energy for Defibrillation

PROTOCOL

A feasibility study of
Prehospital Optimal Shock Energy for Defibrillation (POSED)

ISRCTN Number:	ISRCTN16327029
Sponsor:	University of Warwick
Funding Body:	NIHR ICA Clinical Doctoral Research Fellowship
Ethics Approval date:	London (Harrow) 19 Feb 2021
Version Number:	5.0
Date:	12 Dec 2022
Stage:	Final

This protocol has regard for current HRA guidance and content

Protocol Amendments:

**Amendment No.**	**Date of Amendment**	**Date of Approval**
1.1	23 Jun 2020	
1.11	17 Sep 2020	19 Feb 2021
2.0	15 Apr 2021	08 Jun 2021
3.0	10 Nov 2021	10 Jan 2022
4.0	28 Jul 2022	27 Jul 2022
5.0	12 Dec 2022	14 Dec 2022

WARWICK CLINICAL TRIALS UNIT

WARWICK THE UNIVERSITY OF WARWICK

NIHR | National Institute for Health Research

CONTACT NAMES AND NUMBERS

Role	Name, address, telephone
**Sponsor:**	Carole Harris<br>Head of Research Governance<br>Research & Impact Services<br>University House<br>University of Warwick<br>Coventry<br>CV4 8UW<br>Tel: 02476 522746<br>Email: sponsorship@warwick.ac.uk
**Chief Investigator:**<br><br><br><br><br><br><br><br><br>**Co-Chief Investigator:**	Prof Gavin Perkins<br>Warwick Clinical Trials Unit<br>University of Warwick<br>Gibbet Hill Campus<br>Coventry CV4 7AL<br>Tel: 02476150925<br>Fax: 02476151136<br>Email: g.d.perkins@warwick.ac.uk<br><br>Helen Pocock<br>Warwick Clinical Trials Unit<br>University of Warwick<br>Gibbet Hill Campus<br>Coventry CV4 7AL<br>Tel: 02476 715173<br>Fax: 02476151136<br>Email: helen.pocock@warwick.ac.uk
**Study Co-ordinator:**	Jeskaran Rai<br>Warwick Clinical Trials Unit<br>University of Warwick<br>Gibbet Hill Campus<br>Coventry CV4 7AL<br>Tel: 02476573369<br>Fax: 02476151136<br>Email: jeskaran.rai@warwick.ac.uk

Role	Name, address, telephone
**Co-investigator:**	Prof Charles Deakin<br>South Central Ambulance NHS Foundation Trust<br>Sparrowgrove<br>Southern House<br>Otterbourne SO21 2RU<br>Tel: 01962 898071<br>Email: Charles.deakin@scas.nhs.uk
**Statistician:**	Prof Ranjit Lall<br>Warwick Clinical Trials Unit<br>University of Warwick<br>Gibbet Hill Campus<br>Coventry CV4 7AL<br>Tel: 02476574659<br>Fax: 02476151136<br>Email: r.lall@warwick.ac.uk
**Study Oversight Committee:**	Chair: Prof Tom Quinn<br>Faculty of Health, Social Care and Education,<br>Kingston University & St George's, University of London,<br>Cranmer Terrace<br>London SW17 0RE<br>Tel: 0208 725 4229<br>Email: t.quinn@sgul.kingston.ac.uk
**PPI representatives:**	Miss Debra E Smith<br>c/o Study Co-ordinator<br><br>Mrs. Anne Devrell<br>c/o Study Co-ordinator
For general queries and supply of study materials please contact the coordinating centre:<br>Warwick Clinical Trials Unit (WCTU)<br>The University of Warwick<br>Gibbet Hill Road<br>Coventry<br>CV4 7AL<br>Tel: 02476573369<br>Fax: 02476151136	

TABLE OF CONTENTS **PAGE**

TABLE OF CONTENTS 4
STUDY SUMMARY 7
LIST OF ABBREVIATIONS/GLOSSARY 8
1. BACKGROUND 10
1.1 Epidemiology and burden of the condition 10
1.2 Existing knowledge 10
1.3 Hypothesis 12
1.4 Need for a trial 13
1.5 Ethical considerations 13
1.6 CONSORT 16
1.7 Assessment and management of risk 16
2. STUDY DESIGN 17
2.1 Study summary and flow diagram 17
2.2 Aims and objectives 18
2.2.2 Secondary objective 18
2.3.1 Feasibility outcomes 20
2.3.2 Clinical outcomes 20
2.3.3 Process outcomes 22
2.3.4 Safety 23
2.4 Eligibility criteria 23
2.4.1 Inclusion criteria 23
2.5 Participant identification / Screening 23
2.6 Site Staff Information 24
2.7 Informed consent 24
2.8 Randomisation 28
2.8.1 Randomisation 28
2.8.2 Post-randomisation withdrawals, exclusions and Moves out of Region 29
2.9 Study treatments / intervention 29
2.9.1 Study treatment(s) / intervention 29
2.9.2 Compliance/contamination 30
2.10 Blinding 30
2.10.1 Methods for ensuring blinding 30
2.10.2 Methods for unblinding the study 30
2.11 Co-enrolment to other studies 30

2.12 End of study ... 30
3. METHODS AND ASSESSMENTS ... 31
3.1 Schedule of delivery of intervention and data collection ... 31
3.2 Long term follow-up assessments ... 33
3.3 Assessment of approach to informing relatives of non-survivors ... 33
3.4 Qualitative assessments – Nested studies ... 33
3.4.1 Aims and objectives ... 34
3.4.2 Sample ... 34
3.4.3 Recruitment and consent ... 34
3.4.4 Intervention ... 34
3.4.5 Data management and analysis ... 35
4. ADVERSE EVENT MANAGEMENT ... 35
4.1 Definitions ... 35
4.1.1 Adverse Events (AE) ... 35
4.1.2 Serious Adverse Events (SAEs) ... 35
4.2 Reporting SAEs ... 36
4.3 Responsibilities ... 38
4.4 Notification of deaths ... 39
4.5 Reporting urgent safety measures ... 39
5. DATA MANAGEMENT ... 40
5.1 Data collection and management ... 40
5.2 Database ... 42
5.3 Data storage ... 42
5.4 Data access and quality assurance ... 42
5.5 Data Shared with Third Parties ... 43
5.6 Archiving ... 43
6. STATISTICAL ANALYSIS ... 44
6.1 Power and sample size ... 44
6.2 Statistical analysis of efficacy and harms ... 44
6.2.1 Statistics and data analysis ... 44
6.2.2 Planned recruitment rate ... 44
6.2.3 Statistical analysis plan ... 45
6.2.3.1 Summary of baseline data and flow of patients ... 45
6.2.3.2 Primary outcome analysis ... 45
6.2.3.3 Secondary outcome analysis ... 45
6.3 Interim analysis and criteria for the premature termination of the study ... 46
6.4 Subject population ... 47
6.5 Procedure(s) to account for missing or spurious data ... 47

7. STUDY ORGANISATION AND OVERSIGHT ... 48
7.1 Sponsor and governance arrangements ... 48
7.2 Ethical approval ... 48
7.3 Study Registration ... 49
7.4 Notification of serious breaches to GCP and/or study protocol ... 49
7.5 Indemnity ... 49
7.6 Study timetable and milestones ... 50
7.7 Administration ... 50
7.8 Study Management Group (SMG) ... 50
7.9 Study Oversight Committee (SOC) ... 50
7.10 Data Monitoring Committee (DMC) ... 51
7.11 Essential Documentation ... 51
7.12 Financial Support ... 51
8. MONITORING, AUDIT AND INSPECTION ... 51
9. PATIENT AND PUBLIC INVOLVEMENT (PPI) ... 52
10. DISSEMINATION AND PUBLICATION ... 53
11. REFERENCES ... 54

LIST OF TABLES PAGE

No table of figures entries found.

LIST OF FIGURES PAGE

Figure 1 Study flow diagram ... 19
Figure 2 Pathway to survival following successful defibrillation ... 22
Figure 3 Strategies for informing participants/relatives ... 26

STUDY SUMMARY

Study Title	A feasibility study of Prehospital Optimal Shock Energy for Defibrillation	
Short title	POSED	
Study Design	Single site three-armed parallel-group feasibility randomised controlled trial	
Study Participants	Adult patients sustaining out-of-hospital cardiac arrest attended by a crew from the participating ambulance service in whom resuscitation is attempted and a shock indicated.	
Planned sample size	90 (30 in each arm)	
Treatment Duration	Duration of cardiac arrest (up to approximately 60-minutes)	
Follow-up Duration	To hospital discharge and 30-days	
Planned Study Period	From Sep 2020 to Nov 2022	
	**Objectives**	**Outcome Measures**
Primary	Establish whether it is feasible to conduct a large scale definitive trial by establishing the number of eligible patients and the number recruited.	Eligibility screening logs based on Ambulance Service call data and clinical records.
Secondary	1. To measure the rate of adherence to the allocated treatment.<br><br>2. Identify the best outcome measures in terms of ease and reliability of recording by reviewing data completeness	Rate of compliance with treatment allocation<br><br>Rate of shock data capture on clinical records; Rate of Rankin Focussed Assessment (RFA) completion
**Sub-studies**	**Objectives**	**Outcome Measures**
Qualitative	Exploration of views and experiences of paramedics regarding recruitment, treatment adherence and data completeness	Analysis of focus groups data

LIST OF ABBREVIATIONS/GLOSSARY

Abbreviation	Explanation
AE	Adverse Event
AED	Automated External Defibrillator
BTE	Biphasic Truncated Exponential waveform
CAD	Computer Aided Despatch
CCU	Coronary Care Unit
CI	Chief Investigator
CONSORT	*Con*solidated *S*tandards *o*f *R*eporting *T*rials
CRF	Case Report Form
CTU	Clinical Trials Unit
DMC	Data Monitoring Committee
ED	Emergency Department
ePR	Electronic Patient Record
ERC	European Resuscitation Council
GCP	Good Clinical Practice
HDU	High Dependency Unit
ICF	Informed Consent Form
ICU	Intensive Care Unit
ILCOR	International Liaison Committee On Resuscitation
IRAS	Integrated Research Application System
ISRCTN	International Standard Randomised Controlled Trial Number
J	Joules
MRC	Medical Research Council
PI	Principal Investigator
PPI	Patient & Public Involvement
pVT	Pulseless Ventricular Tachycardia
QA	Quality Assurance
QoL	Quality of Life
RCT	Randomised Controlled Trial
REC	Research Ethics Committee
RFA	Rankin Focussed Assessment
RLB	Rectilinear Biphasic waveform
R&D	Research and Development
SAE	Serious Adverse Event
SOC	Study Oversight Committee

SOP	Standard Operating Procedure
ToF	Termination of Fibrillation
VF	Ventricular Fibrillation
VT	Ventricular Tachycardia
WCTU	Warwick Clinical Trials Unit
WMS	Warwick Medical School

1. BACKGROUND

1.1 Epidemiology and burden of the condition

Cardiac arrest, the cessation of the pumping action of the heart, is one of the primary causes of premature death across the world.[1] A lack of mechanical activity of the heart may be accompanied by a number of different electrical states, broadly divided into 'shockable' and 'non-shockable' rhythms. The shockable rhythms, ventricular fibrillation and (pulseless) ventricular tachycardia, may be characterised by the rapid and chaotic passage of electrical signals through the heart resulting in a lack of co-ordinated pumping action. Cessation of this chaotic activity is an essential precursor to restoration of a perfusing rhythm.[2] A defibrillator is used to deliver shocks to the myocardium by rapidly discharging energy through electrodes applied to the chest; *defibrillation* has been achieved when sufficient current has been delivered to the myocardium to cease the propagation of chaotic electrical impulses.[3]

The vast majority of cardiac arrests occur in the pre-hospital environment and their treatment remains a community health challenge.[4]

In 2014, approximately 30,000 resuscitation attempts were made by UK emergency medical services (EMS). Of these, 6,000 (20%) were in an initially shockable rhythm and, of these, 20.9% survived to hospital discharge.[5]

Key variables in shock delivery are the waveform, shock energy and delivery protocol. There are two different waveforms in common use, determined by the manufacturer of the defibrillator. The biphasic truncated exponential (BTE) waveform delivers a peak current which decays exponentially before reversing direction. The rectilinear biphasic (RLB) waveform maintains current at a roughly fixed level of current in a saw-tooth waveform before reversing direction. There is currently no clear consensus on optimal shock energies and no evidence that one waveform is better than another; European Resuscitation Council (ERC) guidelines advising both to treat with an initial energy of at least 150J for biphasic waveforms, escalating after a failed shock if the defibrillator is capable, and to base shock energy on manufacturers' guidance.[6] The International Liaison Committee on Resuscitation (ILCOR) has identified defibrillation energy levels as a priority area for research.[7]

1.2 Existing knowledge

There is a lack of evidence for optimal first-shock energy level and no strong evidence favouring either fixing subsequent shocks at the same level or escalating the energy.[7] Delivery of too little

energy is unlikely to defibrillate the heart whilst too much may cause myocardial injury, manifested by conversion to a non perfusing rhythm, such as asystole or ventricular arrhythmias (e.g. refibrillation).[8] Studies have identified that lower energy shocks result in less damage to the myocardium than higher energy shocks.[9]

If the shock energy is right first time, the resuscitation duration is shortened, and circulation restored more quickly. Amongst witnessed cases, the chance of survival to 30 days decreases with each defibrillation (OR 0.9; 95% CI 0.88-0.92).[10] The duration of the resuscitation attempt, measured from initiation to return of spontaneous circulation (ROSC), also affects neurological outcome. The chance of surviving with good neurological outcome at one month is significantly higher in those achieving ROSC within 5 minutes compared to those who achieve ROSC after more than 31 minutes (AOR 0.04; 95% CI 0.03-0.05).[11] It is important to shorten the resuscitation duration by delivering the optimal shock energy on first and subsequent shocks.

Patient populations requiring defibrillation in the community display different characteristics from those in a hospital setting. Generally, patients sustaining cardiac arrest out-of-hospital have fewer pre-existing co-morbidities and are more likely to sustain unwitnessed arrests. There is usually a significant delay in getting a defibrillator and professional help to the patient whereas there is rapid access to the team, equipment and information in the hospital setting.[12] A trial assessing optimal defibrillation strategy is required in the pre-hospital setting. This would involve identifying the best first shock energy and whether subsequent shocks should be delivered at the same or a higher energy.

1.2.1 First shock energy

Although several studies report first shock success, none has provided sufficient strength of evidence on which to base treatment recommendations. A previous systematic review reported no observed difference in first shock success for energy levels between 120-200J.[9] However, this was based on evidence published prior to 2011, when there was less emphasis on good quality chest compressions and shocks were delivered in stacks of three. Only two studies employ 2010 CPR guidelines, with its emphasis on early defibrillation and single shocks separated by high-quality CPR.[13, 14] The first of these compares manual versus automated delivery of BTE 360J shocks, where fibrillation was terminated in 80.7 - 84.3% of cases.[13] The second paper does not report energies and employs both biphasic waveforms, making no comparison of outcomes between them.[14]

1.2.2 Fixed versus escalating strategy

In a fixed regime, shock energy remains constant throughout the resuscitation attempt; an escalating regime increases the energy above that of the first shock. The latest ILCOR Consensus on Science statement advised that if the first shock is unsuccessful it is reasonable to escalate the energy for subsequent shocks.[7]

In the BIPHASIC trial, an escalating high-energy protocol was more successful than the fixed low-energy protocol at both terminating fibrillation (81.8% vs 71.8%) and restoring an organised rhythm (36% vs. 25.7%).[15] However, a stacked shocks regime was employed, and the study was also underpowered to detect a difference in survival. A recent post-hoc cohort analysis of the CIRC trial, which compared manual and mechanical chest compression delivery, explored the effect of shock strategy. No difference in long-term survival was detected between patients receiving escalating energy shocks (200-300-360J) and those receiving a fixed high-energy shocks (360-360-360J) but patients in the high-energy group received more shocks and more drugs during their resuscitation attempt.[16] This may be due to an interaction effect or because the higher energy shocks caused myocardial stunning, but no strong conclusions can be drawn due to the post-hoc non-randomised nature of the analysis.

The evidence base for first shock energy is weak as is that for subsequent shock energies; much of the evidence is based on old resuscitation regimes and uses a variety of different endpoints making meaningful comparison difficult.[9] There is a clear need to ascertain (1) optimal first shock energy and (2) subsequent shock strategy, to improve outcomes following cardiac arrest.

1.3 Hypothesis

It is important to establish whether paramedics would be willing to deliver a randomised intervention. Previous cardiac arrest research has failed due to a lack of paramedic engagement.[17] Beliefs about patient benefit relating to the randomisation of treatments have previously concerned paramedics and may preclude their involvement in the project.[18]

The main research question is:

Is it feasible to conduct a randomised, pragmatic clinical effectiveness trial in UK ambulance services to identify the optimal energy for defibrillation?

1.4 Need for a trial

In the absence of definitive evidence, UK Ambulance Services currently employ a range of initial shock energies and differing strategies for subsequent shocks (fixed versus escalating energy). The trial that this feasibility study would inform would provide the evidence for an optimal regime thereby standardising UK (and international) practice.

Such a trial has the potential to impact a significant number of patients per year. Of the 30,000 UK out-of-hospital cardiac arrests per annum, if 20% are in shockable rhythms and survival amongst this group can be increased by 10% (i.e. 20% to 22%) an additional 120 lives per year could be saved.

1.5 Ethical considerations

The study will be conducted in full conformance with the principles of the Declaration of Helsinki and to Good Clinical Practice (GCP) guidelines. It will also comply with the Mental Capacity Act 2005 and Warwick Standard Operating Procedures (SOPs). All data will be stored securely and held in accordance with Data Protection Act 2018.

The Mental Capacity Act sets out criteria by which it may be lawful to recruit adult study participants who lack capacity. This applies if the research cannot be carried out in participants who have capacity, and is subject to approval by an appropriate ethics committee.[19]

The ethical issues raised by this study have been further considered in accordance with the template produced following a HRA-hosted workshop on research involving a waiver of consent or consultation.[20]

1.5.1 Is this research necessary and is there uncertainty regarding treatment?

When a person's heart stops beating the pumping action of the heart is lost and the circulation of blood around the body ceases. The brain may survive for three to four minutes without oxygen and nutrients delivered by the blood and so every second counts when treating cardiac arrest. Successful treatment depends on prompt recognition that the patient is sustaining cardiac arrest, early call for help, prompt commencement and continuation of good quality chest compressions (CPR) and early defibrillation where appropriate. But even the best quality CPR can only provide one third of the effectiveness achieved by the heart in terms of cardiac output and cerebral perfusion. Hence restoration of a heartbeat is a time-critical emergency.

It is known that defibrillation improves patients' chances of survival and the earlier a shock can be delivered the greater the chance of survival.[10, 21] Survival rates of 20.9% have been achieved where a shock was delivered following a witnessed cardiac arrest compared with 7.2% where the rhythm was non-shockable.[5] Rates as high as 74% of patients have been achieved when a shock has been delivered within 3 minutes of collapse.[21] However, current evidence is unclear regarding the best energy level to deliver; too much energy risks damage to the heart whereas too little risks failing to defibrillate. The International Liaison Committee on Resuscitation (ILCOR) have identified the need to address this knowledge deficit as a research priority.[7]

1.5.2 Is it necessary to recruit patients who lack capacity and might patients' capacity fluctuate during treatment?

When a person sustains a cardiac arrest, the effects on their level of consciousness are almost instantaneous. They collapse unconscious and therefore lack capacity. This would continue to be the case throughout the intervention period. There is no alternative group of patients in whom this intervention can be tested.

1.5.3 Does the treatment need to be given quickly and how might the patient be affected by delay?

Due to the life-threatening nature of the condition, cardiac arrest needs to be treated as quickly as possible. Without treatment the patient will not survive and a patient's chances of survival reduce by 7-10% with each minute of delay to resuscitation.[22] The study design ensures that there will be no interruption to delivery of defibrillation, CPR or other elements of resuscitation, which will be delivered in accordance with current UK Resuscitation Council guidelines.[23]

The Study Oversight Committee will monitor safety by tracking trends in survival.

1.5.4 Is consent or consultation possible in this situation?

During cardiac arrest patients will be unconscious and therefore lack capacity. It is not possible therefore to seek their consent in this situation. Due to the time critical need for treatment, it is impractical to consult a personal or professional consultee without causing harmful delays to the treatment of the patient.

1.5.5 What will patients or their consultee be asked later?

A condition of granting deferred consent is that the patient or their consultee must be informed about the study and their consent to continue in the study sought at the earliest opportunity.[25] The early phase of cardiac arrest treatment is usually a time of heightened emotion for relatives.

During this time asking them to process complex information about the study and give consent on their relative's behalf is likely to result in unnecessary additional burden. The most appropriate time to discuss the study with survivors and relatives will balance the need for emotional sensitivity with legal requirements. We will approach survivors when the 'initial emergency' phase is over: when they have sufficiently recovered to leave critical care and move to a ward. The first information provided to patients about their enrolment will be via face to face meeting. This is still a very difficult time for the patient and their relatives, and face to face interaction helps to demonstrate the requisite respect and sensitivity. If patients do not regain capacity, attempts will be made to identify a consultee (either personal or nominated) to provide information about the study and the patient's enrolment, and to seek their opinion as to whether the patient would have any objection to taking part in the follow-up phase of the study (survival status and Modified Rankin Scale (mRS) scoring at 30 days). Our experience with the PARAMEDIC2 trial tells us that this strategy is acceptable both to patients and their relatives.[26]

1.5.6 Informing relatives of non-survivors about the study

Unfortunately, most patients sustaining out-of-hospital cardiac arrest will not survive. At this difficult time, any unnecessary emotional burden ought to be avoided but openness about the study may prevent further upset later. Sensitive consideration must be made of the best means of informing relatives of non-survivors. We need to balance openness with the burden placed on relatives of non-survivors by informing them of their relative's study participation. We have engaged extensively both with patient and public groups and with researchers from around the world in deciding how best to approach this issue. We have broad support for actively engaging with relatives following patient enrolment although opinion was divided regarding the timing of the approach. Our consultees gave a strong steer away from provision of clinical and research information in this first contact, which may be difficult to understand, and instead, towards supportive strategies that may help relatives to come to terms with their loss. Links to, and opportunities for, accessing further information will be made available in our communication.

1.5.7 Patient identifiable data

No personal identifiable data will be held by the study coordinating centre. Personal identifiable data will be shared between the Ambulance service and the receiving hospital for the purpose of follow-up to hospital discharge and between the Ambulance service and the patient's GP for the purpose of informing the GP of the enrolment and follow-up if consent is granted.

1.6 CONSORT

The study will be reported in line with the updated CONSORT (*Con*solidated *S*tandards *o*f *R*eporting *T*rials) statement (British Medical Journal 2010, **340**).

1.7 Assessment and management of risk

A risk assessment will be undertaken to detail the potential hazards of the study and subsequent monitoring will be performed in line with Warwick CTU's monitoring procedures.

2. STUDY DESIGN

2.1 Study summary and flow diagram

POSED is a stand-alone open-label feasibility study. A single-centre, cluster-randomised multi-arm study will be performed to assess feasibility measures that would inform the design of a large-scale trial. This single-centred study will be hosted by South Central Ambulance Service, an NHS ambulance service in the south of England.

The population of interest is patients sustaining out-of-hospital cardiac arrest who, on assessment by an ambulance crew, display a shockable cardiac rhythm (VF or pulseless ventricular tachycardia (VT)). This may be the initial or subsequent rhythm.

Defibrillators will be randomised to deliver one of three shock strategies:

Group	First shock	Second shock	Subsequent shocks	Strategy
1	120	150	200	Escalating
2	150	200	200	Escalating
3	200	200	200	Fixed

The comparator groups have been selected as they represent current UK practice. Group 1 delivers shock energies according to manufacturer's current guidance. Group 2 delivers shock energies according to current practice at the study site, but this is anomalous to the other NHS ambulance services. Although none of the UK ambulance services currently employs the group 3 energies, equivalent energy settings, i.e. high-high-high, are employed using BTE waveform in a number of NHS ambulance services. Group 3 is representative of current practice in these ambulance services.

In preparation for this study, a small scoping exercise of paramedics was conducted within the proposed study site. The scoping exercise found that 70% of paramedics felt that the first shock should be >120J whilst 95% felt that an escalating regime should be used. Whilst holding opinions on shock strategy, the exercise revealed that 30% of paramedics did not know what energies their defibrillators were programmed to deliver. When asked about the acceptability of randomising the intervention, 95% indicated that they would be willing to take part in a study comparing fixed and escalating shock levels.

Each shock takes a fraction of a second to be delivered by the defibrillator to the patient's heart. Patients may require one or many shocks during the pre-hospital phase. The resuscitation attempt may last from 20 min to approximately 60 minutes depending on journey time to hospital. Study treatment ceases on the handover of care to the hospital team who will continue with their normal care from this point.

The feasibility of assessing the following outcomes for a main trial will be assessed:

- Favourable neurological outcome at discharge and at 30 days (mRS score)
- Return Of Organised Rhythm capable of sustaining a pulse (ROOR) 2 min post (each) shock
- Re-arrest rate (re-fibrillation)
- Survived event (return of spontaneous circulation (ROSC) at hospital handover)
- Survived to hospital discharge and at 30 days

This feasibility study aims to recruit 90 patients, ideally 30 in each arm. This is in keeping with a recommended sample size of at least 50 for feasibility studies.[27]

2.2 Aims and objectives

2.2.1 Primary objective

The primary objective of this study is to establish whether it is feasible to conduct a large-scale definitive trial by establishing the number of eligible patients and the number recruited.

2.2.2 Secondary objective

Secondary objectives of the study are to:

- To measure the rate of adherence to the allocated treatment
- Identify the best outcome measures in terms of ease and reliability of recording by reviewing data completeness.
- Explore what affects treatment adherence and data completeness by eliciting the views and experiences of paramedics via focus groups.

Figure 1 Study flow diagram

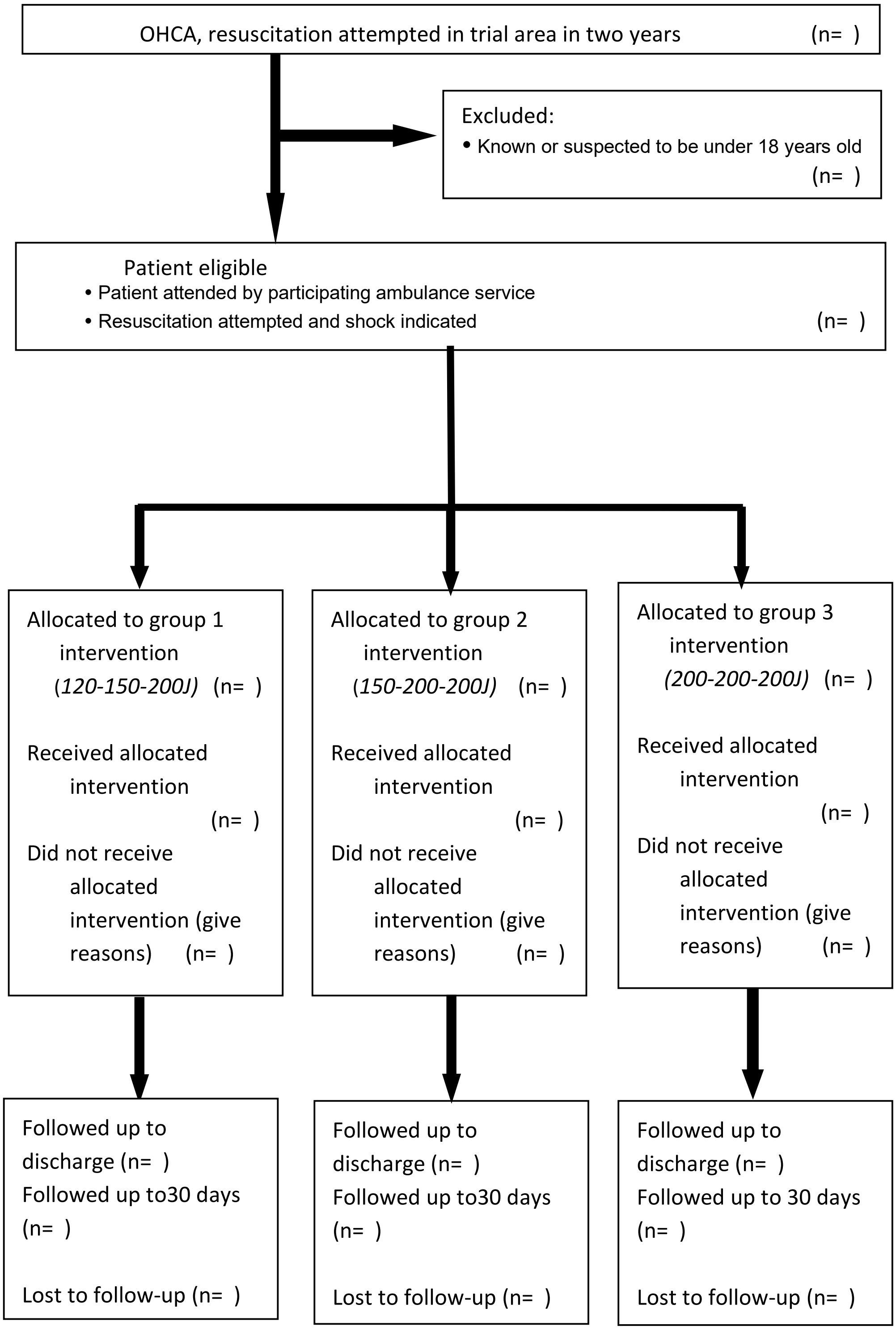

2.3 Outcome measures

2.3.1 Feasibility outcomes

Primary feasibility outcome

- No. of eligible patients and no. of patients recruited

Secondary feasibility outcomes

- Data completeness of clinical outcomes below
- Treatment adherence rate
- Acceptability of approach to informing relatives of non-survivors
- Issues identified by ambulance staff and suggestions for study optimisation.

Eligible patients will be identified from Ambulance service Computer Aided Despatch (CAD) data and Electronic Patient Records (ePR). Data downloaded from defibrillators will indicate the treatment received.

We will monitor the acceptability of our approach to informing the relatives of non-survivors by monitoring the number and nature of enquiries received by the Ambulance service in response to receipt of a letter. Anonymised summaries of each contact will be provided by the Ambulance service Patient Experience Team.

Qualitative data will be gathered from ambulance staff regarding difficulties and suggestions via focus groups. This study will explore the feasibility of collecting the clinical outcome measures, below, for a main trial. The overall aim of a main trial would be to assess the efficacy of various shock strengths.

2.3.2 Clinical outcomes

Outcomes, their time of measurement and source are shown in table 1.

Outcome	Time point of measurement	Data Source
Return Of Organised Rhythm (ROOR)	2 minutes after delivery of each shock	Defibrillator data downloads
Resulting rhythm (VF/pVT/PEA/asystole)	2 minutes after delivery of each shock	Defibrillator data downloads
Refibrillation rate	At any point during out-of-hospital phase	Defibrillator data downloads

Survived event (return of spontaneous circulation (ROSC) at hospital handover)	At hospital handover	Patient status at handover recorded on electronic patient clinical record by ambulance staff
Survival to 30 days and hospital discharge	30 days and hospital discharge	Hospital patient records/ GP patient records
Neurological function (mRS score) at 30 days and hospital discharge	30 days and hospital discharge	Assessment of patient and/or clinical records by research paramedic/GP to determine mRS score

Table 1: Clinical outcomes

The ultimate aim of cardiac arrest treatment is to achieve survival with good neurological function and this is one of the prioritised outcomes making up the consensus-derived core outcome set for cardiac arrest (COSCA).[28] However, an important component of assessment of treatments for cardiac arrest in which the rhythm is shockable is establishing shock efficacy. Key steps in the pathway following successful defibrillation are shown below:

Figure 2: Pathway to survival following successful defibrillation

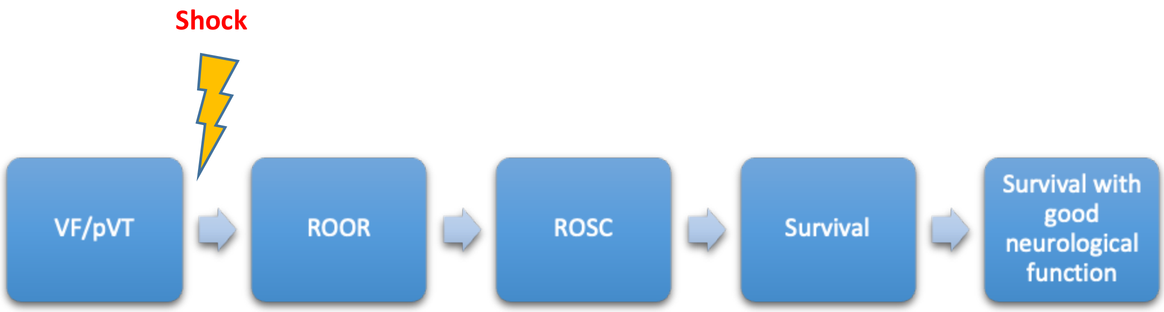

VF = ventricular fibrillation pVT = pulsed ventricular tachycardia ROOR = return of organised rhythm ROSC = return of spontaneous circulation

Traditionally, shock success was defined as the ability of a shock to cease the chaotic electrical activity within the heart, which underlies the mechanical state of fibrillation, within five seconds of shock delivery.[29] However, termination of fibrillation (ToF) is not a good measure of shock success since the termination of all electrical activity (i.e. asystole) would be regarded as a successful outcome whereas this is the worst possible clinical outcome. The Return of Spontaneous Circulation (ROSC) would be a preferred patient-focussed outcome but is problematic since it is

difficult to reliably detect in the prehospital setting.[30] ROSC is usually indicated by the manual detection of a carotid pulse. However, when circulation is first re-established the pulse is often weak and healthcare providers have low confidence in their ability to detect weak pulses.[31, 32] In such cases ROSC may be misclassified as Pulseless Electrical Activity (PEA).[33] The in-hospital practice of verifying ROSC using end-tidal carbon dioxide monitoring is not currently routine prehospital practice in the UK.[34] Although possible to confirm ROSC using such monitoring at the time of hospital handover this time point would not provide a pure indicator of shock success due to possible confounding by post-shock treatments.

Return of organised rhythm (ROOR), defined as the detection of two QRS complexes <5s apart, offers a sensitive marker of shock success. Its presence can be assessed by analysis of cardiac rhythm data recorded by defibrillators during guideline-specified pauses in CPR. These pauses occur two minutes post-shock and allow rescuers to analyse a cardiac rhythm in order to determine whether they should deliver a shock. A recent observational study found that these two-minute rhythm checks could provide valuable prognostic information and identified ROOR as the best prognostic marker for survival when compared to VF or asystole.[35] If ROOR is not achieved it may be useful to know the resultant rhythm to assess whether too little (VF/pVT) or too much (asystole) energy may have been delivered and hence which side of the dose-response curve the energy level falls.[36]

The secondary outcomes, survival and neurological function at 30 days, are distal clinical markers of shock success but are the COSCA-recommended outcome measures of resuscitation success.[28] These outcomes can be reported either at discharge or 30 days, in accordance with the Utstein cardiac arrest reporting template, but reporting of a composite outcome (i.e. 'discharge or 30 days') is discouraged. [28, 37] The modified Rankin Scale (mRS) is an adapted version of a scale used to assess level of disability following non-fatal stroke.[38] It is an ordinal scale scoring level of disability from 0 (no symptoms) to 6 (dead) which may be conducted through face to face or telephone interview of the patient or informant.[39] Where this is not possible, trained health professionals may reliably administer the rating through assessment of hospital notes.[39]

2.3.3 Process outcomes

Process outcomes, including CPR metrics and resuscitation treatments, will be reported. These will be obtained from the defibrillator data downloads and electronic patient record. The following process outcomes will be recorded:

- Quality of CPR (chest compression rate, chest compression depth, chest compression fraction, pre-shock pause, post-shock pause)

- Number of shocks

- Advanced airway applied (% advanced airway applied and % supraglottic airway or endotracheal tube)

- Intravenous medicines administered (% cases where medicines administered and % adrenaline, amiodarone)

- Transported to hospital (% transported)

2.3.4 Safety

In addition to the reporting of outcomes, the system for reporting adverse events and serious adverse events by ambulance service staff will follow the WCTU SOP (see section 4).

2.4 Eligibility criteria

Patients are eligible to be included in the study if they meet the following criteria:

2.4.1 Inclusion criteria

1. Patients sustaining OHCA attended by a crew from participating ambulance service
2. Resuscitation attempted and shock delivered as per Resuscitation Council UK and JRCALC guidelines

2.4.2 Exclusion criteria

1. Patients known or suspected to be under 18 years old

Since the study is not powered to answer a clinical question about this specific group, we consider that the burden of follow-up to these patients and/or family would outweigh any potential benefits and so they will be excluded from the study.

Existing resuscitation protocol advises an initial shock energy of 150J, or to follow manufacturer's guidance.[6] The study protocol is therefore consistent with existing resuscitation protocols.

2.5 Participant identification / Screening

The intervention will be delivered by ambulance staff attending patients sustaining cardiac arrest. 'Ambulance staff' refers to a range of staff grades normally sent to treat cardiac arrest and including paramedics, technicians, associate ambulance practitioners, emergency care assistants, ambulance nurses, specialist paramedics, and doctors. In this protocol the term 'staff member' will be used to denote any of these grades. Resuscitation is commenced, if appropriate, according to Joint Royal College Ambulance Liaison Committee (JRCALC) guidelines.

Patients receiving initial shocks from non-ambulance service defibrillators (e.g. public access defibrillators) may be included in the study. A patient may be eligible, but not recruited, if the incident was attended by a POSED vehicle carrying its allocated defibrillator, but shocks were delivered by an ambulance service non-POSED defibrillator.

Recruitment rates will be monitored via ambulance service call and clinical record databases. Screening logs will be kept listing potentially eligible patients who are not recruited.

2.6 Site Staff Information

Study-specific information will be delivered to ambulance staff providing the study intervention including:

- Background and rationale for study
- Ethical issues and consent
- Overview of GCP principles and specific study-related elements
- Study design
- Data collection and documentation

2.7 Informed consent

Based on the rationale in section 1.5, permission to take deferred consent for continuation in the study will be sought from the ethics committee. Conducting emergency research requires the building and maintenance of public trust and a sensitive approach to information sharing.[40, 26] Developing public awareness of a study has been found to reassure those recruiting patients into deferred consent studies of the acceptability of this approach.[41] Decisions regarding the level and timing of the receipt of information will vary according to a person's relationship to the study. For example, the relatives of a non-surviving participant may have different informational needs and capacity to process information from those of a surviving participant.

The diagram below outlines the general approach to the communication of study information.

Figure 3: Strategies for informing participants/relatives/professional consultee

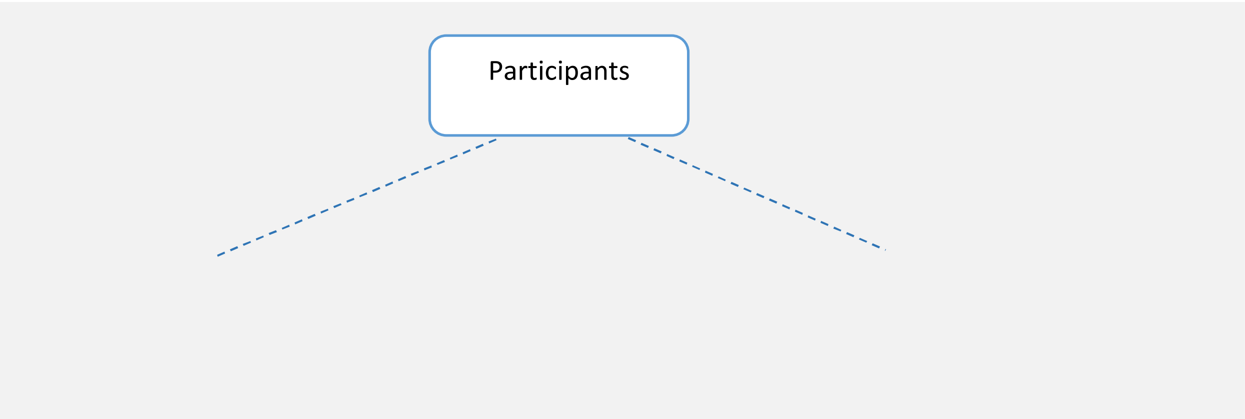

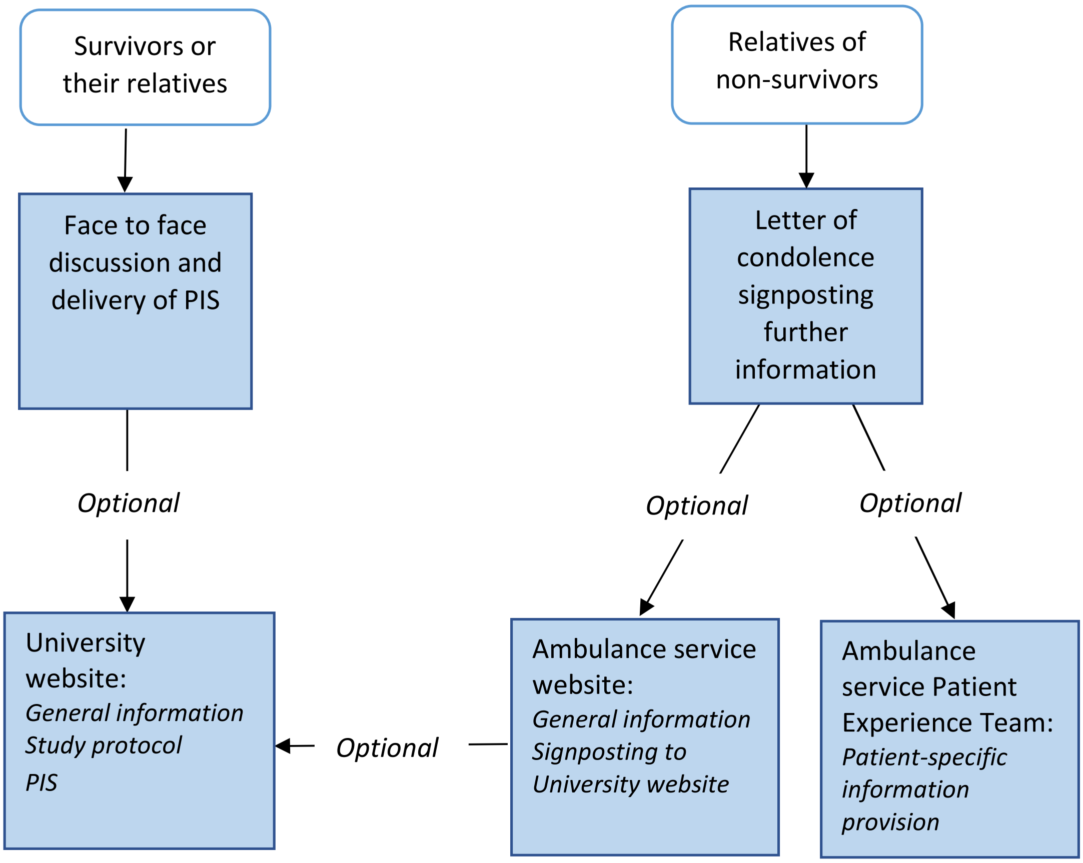

2.7.1 General information regarding the study

We will make available general information about the study on the University website. In addition, general information regarding the study will be made available on the Ambulance service website, public newsletters and through discussion at public meetings. All of these channels will include contact details for further information. In this way members of the public may receive basic information and then choose whether and when to seek further details.

2.7.2 Informing survivors of their inclusion

Survival status of study participants will be tracked via the hospital research teams. Sensitive consideration of the most appropriate time to approach survivors must balance the legal duty to inform with the vulnerable emotional state of survivors and relatives in the early stages of their post cardiac-arrest journey. Our experience with PARAMEDIC2 and discussions with our patient and public advisory panel indicate that the earliest time for this approach is after the initial emergency has passed. This is usually once the patient has left a critical care area (such as the Intensive Care Unit (ICU), High Dependency Unit (HDU) or Coronary Care Unit (CCU) and been moved to a ward. This also allows time for the research team to be made aware of the enrolment, track their location and verify their survival status. A research paramedic will attempt to contact the ward nurse

responsible for the patient's care. This is the healthcare professional who may most quickly develop and maintain rapport with the patient and their family as well as having an ongoing awareness of the patient's mental capacity. Together they will negotiate the most appropriate and earliest visiting time. The research paramedic will meet with patient and, ideally, their family and will first assess the patient's mental capacity. If they have capacity to consent the research paramedic will provide a patient information sheet, discuss the study and inform them of their enrolment. We know from previous experience with the PARAMEDIC2 trial that patients and their families value this contact with paramedics who can also answer any questions regarding the prehospital phase of their care.

Patients shall then be given sufficient time to read through the information, discuss with others and have any questions answered before potentially giving consent.

Patients may decide that they do not wish to discuss the study or to participate. They are free to withdraw from the study at any time without giving reasons and without prejudice to any further treatment. In any of these situations, the patient's decision shall be respected and recorded, and contact details provided should they wish to obtain further information about the study.

In order to keep their care team informed, we will write to the GPs of all recruited patients.

2.7.3 Patients who lack capacity

Where patients lack capacity, we will attempt to contact a personal consultee to ascertain the patient's wishes. Should no personal consultee be available, a nominated consultee shall be approached. An appropriate consultee would be a medical practitioner who is not connected with the study. A similar process to that outlined above shall be followed with identification of a suitable consultee to be discussed with the hospital nursing team. The consultee shall be notified of the patient's enrolment and provided information about the study via the consultee information sheet (CIS). Rather than providing consent, a consultee will be asked for their opinion on whether the patient would have been likely to consent for follow up. If they consider that the patient would have no objection, the consultee will be asked to sign a consultee declaration form.

Prior to conducting follow-up we will attempt to ascertain whether the patient has regained capacity through consultation with the patient's nurse. If the patient is thought to have regained capacity, a meeting will be arranged as above, information provided to the patient and their consent for follow up sought.

2.7.4 Patients who have capacity but require additional support

Interpreters will be sought for patients who do not speak English. If patients are able to give informed consent but are not able to sign the form, we will ask a witness to sign the consent form to confirm that the patient has given verbal consent for follow-up.

2.7.5 Patients who have already been discharged from hospital

In the event that a patient is discharged before we have made contact with them, Research Paramedics will send a letter to them at their discharge address informing them of the study and inviting their continued involvement in the study. Prior to sending the letter confirmation will be sought that the patient has not died by consulting two information sources, the patient's GP and the NHS Summary Care Record.

2.7.6 Obtaining consent

Since the study intervention will already have been given at this point, consent will be sought for follow-up i.e. assessment of their neurological status at 30 days and at hospital discharge. Ideally consent will be sought form the patient, or an opinion from their consultee, on the hospital ward. In exceptional cases, when contact has not been made in hospital, if the patient or their consultee responds to the invitation letter a home visit will be arranged during which consent will be sought. Research paramedics shall manage the consent process. The PI shall ensure that appropriate training is provided to ensure that they are competent to fulfil this delegated responsibility.

The consent form will explicitly list all of the activities and data sources for which consent is sought, allowing the option to choose those for which consent is granted. Data already collected prior to the meeting will be retained unless explicit consent for its use is denied.

2.7.7 Non-survivors

Sadly, most victims of out-of-hospital cardiac arrest do not survive. At the time of death, the study intervention will already have been delivered and no follow up will be conducted and so the provision of consent is not necessary. The aim of any communication with the relatives of non-survivors would therefore be to demonstrate openness and transparency about study participation. This may reduce mitigate any potential future harm that could result from discovering at a later date that their relative had been enrolled in a study without their knowledge. We know from our experience with PARAMEDIC2 that this situation, on the few occasions that it occurred, was extremely distressing for relatives.

We have consulted with our local PPI advisory panel, the University Hospitals Birmingham NHS Foundation Trust PPI (Clinical Research Ambassador Group) group, international researchers and a bereavement lead and patient representative from a local ED regarding the acceptability of informing relatives at this vulnerable time. We have carefully considered their differing views and, on balance, have decided that the best approach would be to inform relatives of the enrolment via a sensitively worded letter. Our advisors felt that the principles of openness and transparency were most important and that by actively engaging with relatives this would preclude possible chance discovery in future which could provoke additional distress and set them back in the bereavement process. Identification of the most appropriate timing of receipt of a letter, again, divided opinion. We have decided that, in common with international practices and the bereavement services of a local hospital, around 4-6 weeks post death would be the optimal time. This would, it is felt, avoid intrusion on the immediate grieving period.

Our PPI advisors have helped us to create a letter of condolence that minimises the burden of information whilst offering support and further information if desired. This approach empowers people to decide whether and when to seek information.

2.8 Randomisation

2.8.1 Randomisation

Due to the time-critical nature of cardiac arrest, delay to shock delivery must be minimised. Any method of individual patient randomisation would necessarily incur a delay as the shock energy would need to be adjusted on the defibrillator. For this reason, randomisation will be on a cluster basis, the unit of randomisation being the defibrillator. Defibrillators are allocated to specific vehicles (ambulances or rapid response cars) and are not usually moved between vehicles as they are paired with the on-board electronic patient record tablet device. The trial initially defined a cluster as a defibrillator, which as noted above, according to ambulance protocols, should remain co-located with a specific ambulance vehicle. During the conduct of the trial, it became evident that, on occasion, defibrillators became separated from their associated ambulance vehicles. As the Ambulance Service tracks vehicles rather than defibrillators it was only possible to track patients where the vehicle and defibrillator remained together. After careful consideration by the Sponsor, with input from the patient and public advisory group and approval from the Research Ethics Committee, the definition of a cluster was clarified as *a defibrillator carried by its associated ambulance vehicle*. To minimise the risk of movement, defibrillator bags will be marked with the callsign of their allocated vehicle and display stickers reminding staff not to move them.

Defibrillators *on their associated ambulance vehicles* will be allocated to one of the three treatment groups in a 1: 1: 1 ratio. The randomisation schedule will be prepared by the study statistician and defibrillators shall be allocated to a treatment arm in a 1: 1: 1 ratio using simple randomisation. The statistician shall ensure that the sequence is not predictable based on vehicle type.

The experimental protocol ends after three shocks. In the event of a change of defibrillator during a patient care episode, normal trust policy will apply regarding shock energy escalation (i.e. second and subsequent shocks to be delivered at 200J).

At the point of device charging, staff will know which treatment they are delivering and so will not be blinded. Possible performance bias will be assessed by monitoring variables such as numbers of patients conveyed to hospital and not conveyed in each of the treatment arms.

Due to the unpredictable nature of out-of-hospital cardiac arrest it is not possible to sequentially allocate ambulances, and hence defibrillators, to events.

2.8.2 Post-randomisation withdrawals, exclusions and Moves out of Region

The treatment allocation determines the energy levels of the first three shocks. Should the patient gain ROSC and then refibrillation occurs, they will receive the next allocated energy level in the allocation.

Should a patient decline consent, the date on which this is communicated shall be recorded on the database and no further contact made. Any data gathered up to this point shall be retained unless the patient has expressly declined consent for this data.

Unless a participant explicitly withdraws their consent, they should be followed-up wherever possible and data collected as per the protocol until the end of the study.

The National Data Opt-Out will not apply to data for this study as only non-identifiable data will be transferred from the NHS to the university.

2.9 Study treatments / intervention

2.9.1 Study treatment(s) / intervention

All participants will receive standard resuscitation treatment according to current Resuscitation Council (UK) and JRCALC guidelines. These advise at least 150J for initial shock and the same or higher energy for subsequent shocks, or to follow defibrillator manufacturer’s guidance. The exception to this is that defibrillation energies will be randomly allocated.

Qualitative sub-study participants shall be invited to attend a focus group session following their normal team meeting on Trust premises.

2.9.2 Compliance/contamination

It will be important to monitor adherence to treatment allocation. Treatment *delivery* will be compared to treatment *allocation*. Should the energy delivery differ from that which was allocated, this shall be deemed non-compliance and will be documented. Treatment delivery will be monitored by retrieving the defibrillator data download.

2.10 Blinding

2.10.1 Methods for ensuring blinding

It is not possible to blind ambulance staff to treatment allocation as they will see the shock energy when they charge the defibrillator. Patients will be blind to treatment allocation due to the clinical nature of cardiac arrest. Control room staff who allocate ambulances vehicles to emergencies will not be aware of the treatment allocation. It is normal practice for the closest available ambulance to be sent to a cardiac arrest incident. Staff treating participants in hospital may be blind to the patient's treatment allocation since the level of defibrillatory shock is not routinely included in the verbal handover. If patients ask what treatment they received they will be asked not to pass this information to the research paramedic as they should complete the modified Rankin Scale (mRS) assessment blind to the treatment allocation.

2.10.2 Methods for unblinding the study

Since this is an open-label study, no unblinding process will be required.

2.11 Co-enrolment to other studies

Co-enrolment with other trials will be reviewed on a case-by-case basis in accordance with national NIHR-supported co-enrolment guidelines. There are many current examples of successful co-enrolment between UK critical care studies, facilitated by these guidelines.

2.12 End of study

The study will end when all participants have completed their hospital discharge and 30 day follow-ups or after 24 months of patient recruitment, whichever is sooner. Within the last month of recruitment, patients will only be followed up to 30 days to enable timely closure of the study.

The study will be stopped prematurely if:

- Mandated by the Ethics Committee

- Following recommendations from the Study Oversight Committee (SOC)
- Funding for the study ceases

The Research Ethics Committee will be notified in writing within 90 days when the study has been concluded or within 15 days if terminated early. A site study closure plan will be developed and acted upon when the last follow up data have been collected and the data cleaned.

3. METHODS AND ASSESSMENTS

3.1 Schedule of delivery of intervention and data collection

3.1.1 Enrolment

Recruited patients' prehospital data will be taken from routinely collected ambulance service data. Patient and event characteristics, Utstein variables, prehospital treatments and initial outcome will be extracted from the patient clinical record. Call time and ambulance response times will be taken from emergency call centre databases.

Defibrillator data may be attached to the patient clinical record. Alternatively, it will be accessed remotely or directly, if necessary, according to local site constraints. Defibrillator data (treatment summary report) can be downloaded from Zoll X-series devices onto USB devices and viewed via the 'Zoll Code Review/Case Review' software. USB devices may be provided for recording data which may then be sent to the study team.

Enrolments will be identified by research paramedics in one of two ways:

1. Automatic reports generated via the electronic patient record (ePR) system.
2. Searching the Clinical Audit Reporting System (CARS) for paper records.

3.1.2 Hospital

Data from hospital records will be obtained through data-sharing agreements between ambulance services and acute trusts. The research paramedic will liaise with the hospital research team and notify them of each enrolled patient handed over to the hospital. Patients treated for out-of-hospital cardiac arrest are normally taken to the Emergency Department (ED) but may be taken directly to a Coronary Care Unit (CCU) or equivalent for cardiac procedures.

For each patient taken to hospital the following data will be sought:

- Missing demographic data not able to be collected by ambulance service
- Survival status
- Date of admission to ITU/HDU/CCU
- Date of admission to the ward
- Date of discharge (if applicable)

Table 2: Study assessments

	Cardiac arrest	Hospital	Day 30
Inclusion/exclusion criteria	✓	x	x
Cardiac arrest data	✓	x	x
Patient identifiers	✓	✓	x
Adverse event reporting	✓	✓	x

Survival checks	✓	✓	x
Survival status	✓	✓	✓
Notification of enrolment and invitation to take part in follow up	x	✓	x
Informed consent	x	✓	x
Neurological outcome (mRS)	x	x	✓

3.2 Long term follow-up assessments

No long-term follow up shall be conducted in this study. The final data collection point will be at 30 days or at hospital discharge, whichever is later, when the mRS scores of those patients willing to take part shall be assessed. This will usually occur in the hospital but in exceptional cases, if the patient has been discharged prior to this point, may take place at an alternative care facility or in the patient's home.

3.3 Assessment of approach to informing relatives of non-survivors

Our approach to informing the relatives of non-survivors has not previously been tested in the UK. We will review how this is received throughout the study by monitoring the number and nature of enquiries received by the Ambulance Service. We will monitor the time taken to identify next of kin, the sources consulted to verify next of kin details and the concordance/discordance between different sources.

3.4 Qualitative assessments – Nested studies

In order to support the quantitative findings from this feasibility study an exploratory sub-study of the experiences of those recruiting patients will be conducted. This will provide qualitative data allowing an understanding of the impact of the study on ambulance staff, enabling decisions regarding a large-scale trial to be taken that maximise its acceptability and effectiveness.[42] A

phenomenological approach will be taken towards understanding the experience of following the study processes and recruiting patients. Since management of cardiac arrest is a team endeavour, we will seek to understand not only the individuals' experience but also the culture within which they operate. We will use focus groups to elicit views and opinions and observe reactions to what is being said.

3.4.1 Aims and objectives

Aim:

To explore and describe the experience of paramedics delivering this study, specifically the barriers and facilitators to patient recruitment.

Objectives:

- To explore strategies for optimising treatment adherence
- To describe problems and possible solutions for improving the ease and reliability of data recording.

3.4.2 Sample

Maximum variation sampling will be employed to elicit a broad range of views from the ambulance staff.[41] We will invite staff, to include those who have and have not treated patients, from geographically disparate teams comprising a variety of clinical grades and length of experience to take part in the focus groups. We will complete 3-4 focus groups, which should allow us to reach data saturation.[43]

3.4.3 Recruitment and consent

Ambulance staff will be offered the opportunity to take part in focus groups mid-way through recruitment which will allow time for the study to bed down.[42]. Those expressing an interest to take part will be sent a Participant Information Sheet. On the day, the focus group facilitator will provide a verbal summary of the PIS before seeking consent. Participants will be asked for their consent to take part in the focus group, audio recording of data, transcription of data, re-contact by a research paramedic should clarification be required and publication of anonymised quotes. It will be made clear that it will not be possible to withdraw consent and have data removed once the focus groups have commenced.

3.4.4 Intervention

Focus groups will be conducted immediately following a team training session, conducted either at the normal Ambulance service premises or via an online platform if the meeting is to happen

remotely. This is the only time when groups are normally together at the same time. This will also provide the context within which people are used to having the practice-related discussions that shape their attitudes and behaviours.[44]

Pre-prepared topic guides will be used to initiate conversation if required. Groups will be audio recorded and transcribed by the researcher.

3.4.5 Data management and analysis

Electronic audio data files will be transcribed by the researcher.

Transcribed and text will be coded supported by NVivo data analysis software.[45] Analysis of the data will be conducted according to the six phases of thematic analysis.[46] Anonymised transcriptions will be stored on secure servers, that are backed up daily, for 10 years at WCTU.

4. ADVERSE EVENT MANAGEMENT

4.1 Definitions

4.1.1 Adverse Events (AE)

An Adverse Event (AE) is defined as any untoward medical occurrence in a participant taking part in healthcare research and which does not necessarily have a causal relationship with this treatment/intervention. A study CRF for the capture of AEs will be developed and reviewed by the CI, statistician and QA team. AEs will be reviewed and monitored by SOC for trends.

AEs occurring from the time of delivery of study treatment until hospital discharge 30 days post treatment (whichever is sooner) should be reported to WCTU via the appropriate CRF.

4.1.2 Serious Adverse Events (SAEs)

A Serious Adverse Event is an AE that fulfils one or more of the following criteria:

- Results in death
- Is immediately life-threatening
- Requires hospitalisation or prolongation of existing hospitalisation
- Results in persistent or significant disability or incapacity
- Is a congenital abnormality or birth defect
- Requires medical intervention to prevent one of the above, or is otherwise considered medically significant by the investigator (e.g. participant safety is jeopardised).

Many of the above-listed SAEs are associated with cardiac arrest and would be expected consequences of cardiac arrest and attempted resuscitation. Only unexpected, related events are reportable.

4.2 Reporting SAEs

Adverse and serious adverse events which are expected in this population of patients will not be reported, if thought to be unrelated to the trial interventions. These should not be reported as SAE. Such conditions include:

- Death
- Is immediately life-threatening
- Hospitalisation
- Persistent or significant disability or incapacity
- Organ failure
- Injuries or complications associated with cardiac arrest or attempted resuscitation

All reportable **SAEs (as defined in section 4.1)** occurring from the time of delivery of study treatment until hospital discharge or 30 days post treatment (whichever is sooner) must be recorded on the SAE Form and sent to the Sponsor **within 24 hours** of the research staff becoming aware of the event. All SAEs must be reported to WCTU within 24 hours of becoming aware of an adverse event that fulfils the criteria for 'seriousness'. Notification may be received via email or telephone. Where full information is not immediately available, verbal reports will be documented and followed up with a written report as soon as possible. On receipt of notification, all SAEs will be reported immediately to the WCTU Quality Assurance (QA) team. A study CRF for the capture and

reporting of SAEs will be developed and reviewed by the CI, statistician and QA team. On receipt of the initial report the QA team will immediately log on the SAE database.

For each **SAE** the following information will be collected:

- full details in medical terms and case description
- event duration (start and end dates, if applicable)
- action taken
- outcome
- seriousness criteria
- causality (i.e. relatedness to intervention), in the opinion of the investigator
- whether the event would be considered expected or unexpected.

Any change of condition or other follow-up information should be uploaded to the study database as soon as it is available. Events will be followed up until the event has resolved or a final outcome has been reached.

The study coordinator will liaise with the investigator to compile all the necessary information. The study coordinating centre is responsible for reporting any related and unexpected SAEs to the sponsor and REC within required timelines.

The causality of SAEs (i.e. relationship to study treatment) will be assessed by the investigator(s) on the SAE form.

**Relationship to study treatment**	**Description**
Unrelated	There is no evidence of any causal relationship
Unlikely to be related	There is little evidence to suggest there is a causal relationship (e.g. the event did not occur within a reasonable time after administration of the study intervention or device). There is another reasonable explanation for the event (e.g. the patient's clinical condition, other concomitant treatment).
Possible relationship	There is some evidence to suggest a causal relationship (e.g. because the event occurs within a reasonable time

	after administration of the study intervention or device). However, the influence of other factors may have contributed to the event (e.g. the patient's clinical condition, other concomitant treatments).
Probable relationship	There is evidence to suggest a causal relationship and the influence of other factors is unlikely.
Definitely related	There is clear evidence to suggest a causal relationship and other possible contributing factors can be ruled out.

The site PI will be responsible for ensuring that SAEs are reported within 24 hours of a member of the research team becoming aware of the event. Events shall be recordable until hospital discharge.

4.3 Responsibilities

The SMG will monitor adverse events on a monthly basis. Cumulative review of all safety information will be conducted by the Study Oversight Committee on a 6-monthly basis. The CI will ensure that independent review of each SAE is conducted.

<u>Principal Investigator (PI)</u>:

1. Using medical judgement in assigning seriousness, causality and expectedness
2. Ensuring that all SAEs are recorded and reported to the Sponsor within 24 hours of becoming aware of the event and provide further follow-up information as soon as available. Ensuring that SAEs are chased with Sponsor if a record of receipt is not received within 2 working days of initial reporting.
3. Ensuring that AEs are recorded and reported to the Sponsor in line with the requirements of the protocol.

<u>Chief Investigator (CI) / Co-chief investigator</u>:

1. Clinical oversight of the safety of patients participating in the study, including an ongoing review of the risk / benefit.
2. Using medical judgement in assigning seriousness, causality and expectedness of SAEs where it has not been possible to obtain local medical assessment.
3. Immediate review of all related and unexpected SAEs
4. Review of specific SAEs in accordance with the study risk assessment and protocol as detailed in the Study Monitoring Plan.
5. Production and submission of annual reports to the relevant REC.

Sponsor:

1. Central data collection and verification of AEs, and SAEs, according to the study protocol.
2. Reporting safety information to the CI, delegate or independent clinical reviewer for the ongoing assessment of the risk / benefit according to the Study Monitoring Plan.
3. Reporting safety information to the Study Oversight Committee (SOC) identified for the study according to the Study Monitoring Plan.
4. Expedited reporting of related and unexpected SAEs to the REC within required timelines.
5. Notifying Investigators of related and unexpected SAEs that occur within the study.

Study Oversight Committee (SOC):

In accordance with the Study Terms of Reference for the SOC, periodically reviewing safety data to determine patterns and trends of events, or to identify safety issues, which would not be apparent on an individual case basis.

4.4 Notification of deaths

Due to the nature of the condition of interest, a high proportion of deaths is expected. Reports of deaths will be made to the SMG every month and reviewed by the SOC every 6 months.

4.5 Reporting urgent safety measures

If any urgent safety measures are taken the CI/Sponsor shall immediately and in any event no later than 3 days from the date the measures are taken, give written notice to the relevant REC of the measures taken and the circumstances giving rise to those measures.

5. DATA MANAGEMENT

A Data Management Plan will be created in accordance with University of Warwick SOPs.

Personal data collected during the study will be handled and stored in accordance with the Data Protection Act 2018 which encompassed the requirements of the EU General Data Protection Regulations and Warwick CTU SOP 15 ‘Data Management’.

For this feasibility study, participants will be identified using a unique study number only. Personal identifiable data will be shared between the Ambulance service and the receiving hospital for the purpose of follow-up to hospital discharge. Outcome data for those patients missed in hospital will be sought by the Ambulance Service from the patient’s GP. Personal identifiable information will therefore be shared between these agencies for this purpose.

5.1 Data collection and management

Source documents, where data are first recorded, include, but are not limited to, Ambulance Service Computer Aided Despatch (CAD) data, Ambulance service Patient Clinical Records (which may be

electronic or paper), hospital records and electronic correspondence. Source data shall be transcribed onto the Case Report Form (CRF) on the study database. The CRFs will be developed by the Chief investigators and the study statistician to collect all required study data.

Data from the ambulance service will be anonymised prior to being entered onto the study database.

The SCAS researcher will be made aware that a patient has been treated for cardiac arrest via one of the two mechanisms as outlined in section 3.1.1. It is anticipated that this should happen within 48 hours of treatment (or 3 days if the cardiac arrest happened over a weekend). Screening log completion will act as a safeguard to ensure that no cases are missed. This should happen within 7 days of cardiac arrest.

Once a case is identified, the SCAS researcher will access the patient's ambulance service clinical record and link this to the CAD data (via the incident number). From these sources, research paramedics will assess whether the patient meets study eligibility criteria and, if so, will transcribe cardiac arrest data, survival status and patient identifiers if the patient is not known to have died. Data from defibrillators will be accessed by the researcher. Quality of CPR measures will be transcribed from this source to the CRF by the researcher. The electrocardiogram (ECG) will be interpreted by the SCAS researcher and checked by a senior clinician. Confirmation of periods of ROSC will be sought from the patient clinical record. Prior to commencement of the study, the SCAS researcher and the senior clinician will review a number of example ECGs to ensure a standardised approach to assessment. Disagreement will be resolved through discussion or referral to a second senior clinician.

Information requests for patients not known to have died will be sent to the appropriate research team at the destination hospital. Information sought will include patient identifiers that the ambulance staff may have been unable to obtain, including survival status, patient location and date of transfer to the hospital ward. This will be sought by completion of a spreadsheet and will be sent securely via nhs.net email system. Ongoing checks regarding patient location and survival status will be made in order that a visit may be arranged at the appropriate time. Personal identifiers will only be shared between those in the direct care team, i.e. the ambulance service and the destination hospital, and not with the WCTU.

The mRS scores of surviving patients will be assessed at 30-days post event by the research paramedics using the Rankin Focussed Assessment (RFA) tool. This tool has excellent inter-observer reliability (unweighted κ of 0.93 (95% CI, 0.85-1.00)).[47] At 30 days, patients may have been

discharged home or they may be on the hospital ward. We will arrange to visit patients in either location to assess mRS. If the patient has been discharged home, two survival checks will first be made before telephoning the patient. Assessing mRS when they have been discharged into their home setting allows patients to accurately assess their abilities regarding their normal activities.[28] However, since the RFA encourages the gathering of information from all available sources it may also be reliably applied in the hospital setting.[47] Should the patient lack capacity at 30 days and remain in hospital the RFA will be completed with the consultee, nursing staff and the medical notes. Should they be at home, the opinion of the patient's consultee will be sought to complete the RFA. Where a proxy is required for mRS assessment, family members have been found to be more reliable than nurses or therapists.[48] A lone working risk assessment shall be carried out in accordance with the Warwick SOP. Home visits shall be carried out within office hours and within daylight hours where possible. Research paramedics shall ensure that they inform a member of the study team prior to entering an address, carry a mobile phone and undertake a dynamic risk assessment during the visit and inform the study team on leaving the address.

All documents will be stored safely in confidential conditions. On all study-specific documents, other than the signed consent, the participant will be referred to by the study participant number/code, not by name. mRS scores will be entered onto the study database and hard copy RFA sheets retained securely at site.

5.2 Database

The database will be developed by the Programming Team at WCTU and all specifications (i.e. database variables, validation checks, screens) will be agreed between the programmer and appropriate study staff.

5.3 Data storage

All essential documentation and study records will be stored by WCTU in conformance with the applicable regulatory requirements and access to stored information will be restricted to authorised personnel.

5.4 Data access and quality assurance

There is no requirement to collect personal information about potential participants. The only details required are call details and an assessment against the eligibility criteria. Information about enrolled participants will be collected by the research paramedics at site. Personal identifiable details will not be required for those patients known to have died. The purpose of gathering personal identifiable data regarding those patients not known to have died is to allow tracking

through hospital and follow up. Data will be gathered from source documents and entered directly onto the study database.

Information regarding patients not known to have died shall be shared between the site study team and the relevant research team in the destination hospital. The minimum data necessary to identify the correct patient and allow follow up shall be shared. This includes full name, date of birth, address, NHS number and GP surgery. Data will be shared between site and the destination on a password-protected spreadsheet via the secure nhs.net system. At the meeting to potentially grant consent, the research paramedic will seek a contact phone number and/or email address for the patient to allow follow up. This will not be necessary if this meeting coincides with the 30-day time point and the patient is still in hospital. Should contact with the patient's GP be required, to acquire outcome data if the patient has left hospital prior to being approached by a research paramedic and has not responded to invitation letters, identifiable information shall be shared with the GP to ensure correct patient identification.

Consent forms shall be securely stored at site with access only available to the site study team. They shall be securely shredded at study closure. Electronic documents shall be stored on the site server in a folder with access restricted to the study team only. Access permissions are granted/revoked by the Trust's IT team to remain secure.

Direct access to source data/documents may be required for study-related monitoring or audit by WCTU.

An electronic copy of the final study data set shall be sent to the participating ambulance service on completion of the study.

5.5 Data Shared with Third Parties

Anonymous, individual participant data that underlie the results reported in this article (text, tables, figures and appendices) will be made available to investigators whose proposed use of the data has been approved by an independent review committee identified for this purpose. Study data will be made available for five years following article publication. Proposals for data access should be directed to G.D.Perkins@warwick.ac.uk. Requestors will need to sign a data sharing agreement.

5.6 Archiving

After the study, site records shall be securely archived in accordance with GCP and retained for ten years. Electronic records shall be stored securely for ten years at site and at WCTU.

6. STATISTICAL ANALYSIS

6.1 Power and sample size

This feasibility study aims to recruit 90 patients, ideally 30 in each arm. This is in keeping with a recommendation of sample sizes of at least 50 for feasibility studies [27] whilst allowing a roughly equal number of patients to be recruiting into each arm. This should be both sufficient to ascertain whether recruitment is feasibly and achievable within a reasonable time scale.

6.2 Statistical analysis of efficacy and harms

6.2.1 Statistics and data analysis

There will not be a formal statistical analysis as the study has not been powered to assess difference in interventions. The analyses will be based on summary statistics, namely mean, standard deviation, median, interquartile ranges and missingness in the data. Where possible 95% confidence intervals will also be given.

6.2.2 Planned recruitment rate

The study aims to recruit 90 patients within a maximum two-year data collection period.

6.2.3 Statistical analysis plan

As this is a feasibility study there will be no interim analyses. However, a statistical analysis plan will be drawn out to illustrate how the data will be assessed, summarised and displayed.

6.2.3.1 Summary of baseline data and flow of patients

Baseline comparability of the randomised groups will be assessed using the following variables:

- Age (median and range)
- Sex (% male)
- Location of arrest (% private residence/ public place/ other)
- Witnessed vs. unwitnessed event (% witnessed and % bystander/ EMS or other healthcare provider/ unwitnessed)
- Bystander CPR vs. no bystander CPR (of those not EMS-witnessed, % provided BCPR prior to EMS arrival)
- Type of initial rhythm (% in VF/pVT, PEA or asystole)
- Time from call to application of defibrillator (median and range)
- Aetiology of cardiac arrest (% cardiac vs. non-cardiac cause)

Flow of patients will be presented using a consort flow diagram (http://www.consort-statement.org/).

6.2.3.2 Primary outcome analysis

The primary objective of this study is to establish whether it is feasible to conduct a large-scale definitive study. We will report the proportion of eligible patients who receive the randomised intervention.

6.2.3.3 Secondary outcome analysis

The secondary outcomes that will be assessed are:

- Treatment adherence rate. This will be assessed in terms of how many patients received the allocated first shock energy and, where more than one shock was delivered, how many received the correct subsequent shock energies.
- Data completeness of clinical outcomes below:
    - Neurologically intact survival at 30 days and hospital discharge (mRS score)
    - Return Of Organised Rhythm (ROOR) 2 min post shock

    - Resulting rhythm (VF/pVT/PEA/asystole) 2 min post shock
    - Re-arrest rate (re-fibrillation)
    - Survived event (return of spontaneous circulation (ROSC) at hospital handover)
    - Survived to hospital discharge and 30 days

  These will be reported in terms of the proportion of patients for whom each of these outcomes was collected.

- Data completeness of process outcomes below:

  - Quality of CPR (chest compression rate, chest compression depth, chest compression fraction, pre-shock pause, post-shock pause)
  - Number of shocks
  - Advanced airway applied (% advanced airway applied and % supraglottic airway or endotracheal tube)
  - Intravenous medicines administered (% cases where medicines administered and % adrenaline, amiodarone)
  - Transported to hospital (% transported)

- Issues identified by ambulance staff and suggestions for study optimisation. These will be reported in terms of the topics identified, with example anonymised quotes, from the thematic analysis of the focus group data.

- Acceptability of approach to informing relatives of non-survivors. We will monitor the number and nature of enquiries received by the Ambulance Service, the time taken to identify next of kin, the sources consulted to verify next of kin details and the concordance/discordance between different sources.

6.3 Interim analysis and criteria for the premature termination of the study

No interim analyses are planned as this is a feasibility study. The study may be terminated prematurely for any of the following reasons:

- Mandated by the Ethics Committee
- The Study Oversight Committee decides that recruitment should cease
- Mandated by the sponsor

- Study funding ceases.

6.4 Subject population

This feasibility study aims to describe the study population in terms of:

- All-treated population: Any subject randomised and eligible for the study that received at least one part of the intervention
- Protocol-compliant population: Any subject who was eligible, randomised and received the protocol required intervention

The treatment of ineligible patients is outlined in section 2.4, above.

6.5 Procedure(s) to account for missing or spurious data

One of the aims of this study is to assess how much data might be missing following all reasonable attempts to collect this data. In order to prevent missing data, the following strategies will be employed:

- Monthly audits of routine ambulance service data will be conducted to ensure that there are no missing eligible cases. A site audit clerk will conduct these checks and report anonymised cases.
- A spreadsheet of data queries will be sent to the ambulance service on a monthly basis
- To maximise follow-up email alerts will be sent to the ambulance service reminding them that a patient has reached their 30-day assessment point

Where data cannot be obtained, reasons shall be recorded on the data queries sheet.

6.6 Sub-groups

In relation to initial shock (VT/pVT) as our outcome, we will conduct sub-group analyses of:

1. Hospital handover (ROSC, CPR ongoing, not conveyed);
2. 30 days survival (died/alive);
3. Survival at discharge (died/alive).

We will assess the interaction of the treatment arm, with each of the sub-groups.

7. STUDY ORGANISATION AND OVERSIGHT

7.1 Sponsor and governance arrangements

The University of Warwick will act as sponsor for this study and University of Warwick's SOPs shall be employed.

7.2 Ethical approval

Application for approval of the study will be made to the Health Research Authority (HRA). All required ethical approval(s) for the study will be sought using the Integrated Research Application System. The study will be conducted in accordance with all relevant regulations.

Before enrolling patients into the study, the study site must ensure that the local conduct of the study has the agreement of the Ambulance Service Clinical Review Group. Recruitment into the study will not commence until all required permissions are obtained and staff have received training. Substantial amendments shall not be implemented until approved by the REC and approved by the participating site.

Annual reports will be submitted to the REC within 30 days of the anniversary date on which the favourable opinion was given, and annually until the study is declared ended. The REC will be

notified of the projected end of the study date either as 90 participants are recruited or after 24 months of patient recruitment. Should the study require premature closure by any party other than the ethics committee listed in section 6.3 the REC shall be notified within 15 days of the mandated closure date. If not halted prematurely, the REC shall be informed within 90 days via the end of study form.

The CI will submit a final report to the required authorities with the results, including any publications within one year of the end of the study.

This protocol has been peer reviewed by Dr. Keith Couper who is an Assistant Professor in Emergency and Critical Care based at WCTU but not involved in this study. Dr. Couper's research interests include cardiac arrest and clinical trials.

Review of the project has also been provided by the NIHR ICA CDRF Interview panel.

7.3 Study Registration

This study will be registered with the International Standard Randomised Controlled Trial Number (ISRCTN) Register.

7.4 Notification of serious breaches to GCP and/or study protocol

A "serious breach" is a breach which is likely to effect to a significant degree –

(a) the safety or physical or mental integrity of the subjects of the study; or
(b) the scientific value of the study

The sponsor will be notified immediately of any case where the above definition applies during the study conduct phase.

The sponsor will notify the research ethics committee in writing of any serious breach of:

(a) the conditions and principles of GCP in connection with that study; or
(b) the protocol relating to that study, as amended from time to time, within 7 days of becoming aware of that breach

7.5 Indemnity

NHS indemnity covers NHS staff, medical academic staff with honorary contracts, and those conducting the study. NHS bodies carry this risk themselves or spread it through the Clinical Negligence Scheme for Trusts, which provides unlimited cover for this risk. The University of

Warwick provides indemnity for any harm caused to participants by the design of the research protocol.

7.6 Study timetable and milestones

	Month	Recruitment
Set-up	1-3	n/a
Recruitment	4-24	90
Qualitative study	12-16	30-40
Follow up	5-25	n/a
Analysis	26-29	n/a

7.7 Administration

The study co-ordination will be based at WMS/WCTU, University of Warwick.

7.8 Study Management Group (SMG)

The Study Management Group, consisting of the project staff and co-investigators involved in the day-to-day running of the study, will meet monthly throughout the project. Significant issues arising from management meetings will be referred to the Study Oversight Committee or Investigators, as appropriate.

7.9 Study Oversight Committee (SOC)

The study will be guided by a group of respected and experienced personnel and trialists as well as at least one 'lay' representative. The SOC will have an independent Chairperson. Face to face meetings will be held at regular intervals, likely to be every six months. Routine business is conducted by email, post or teleconferencing.

The Oversight Committee, in the development of this protocol and throughout the study will take responsibility for:

- Major decisions such as a need to change the protocol for any reason
- Monitoring and supervising the progress of the study
- Reviewing relevant information from other sources
- Informing and advising on all aspects of the study

The membership of the SOC is shown on page 3.

The full remit and responsibilities of the SOC will be documented in the Committee Charter which will be signed by all members.

7.10 Data Monitoring Committee (DMC)

A DMC is not required for this feasibility study. No interim analyses will be conducted. Data will be assessed for completeness rather than analysed for efficacy/effectiveness. Study oversight will be the remit of the Study Oversight Committee.

7.11 Essential Documentation

A Study Master File will be set up according to Warwick SOP and held securely at the coordinating centre.

7.12 Financial Support

The study has been funded by a HEE/NIHR Integrated Clinical Academic Programme Clinical Doctoral Research Fellowship, award number ICA-CDRF-2018-04-ST2-005.

8. MONITORING, AUDIT AND INSPECTION

A Study Monitoring Plan will be developed and agreed by the Study Management Group (SMG) and SOC based on the study risk assessment. Monitoring may be conducted by exploring the study dataset and may include on-site monitoring. In order to assist the sponsor, the ambulance service will develop procedures for internally monitoring the study and to permit direct access to source data during site monitoring visits.

It is not necessary for Ambulance staff participating in the study to complete full GCP training but key elements will be included in the study information package in order to fulfil the HRA recommendation that GCP guidelines are appropriate and proportionate to the study activities undertaken.[49]

Quality assurance checks on eligibility, data collection and consent process will be carried out by a research paramedic (not the researcher) using a WCTU remote monitoring checklist at least once during the recruitment period.

9. PATIENT AND PUBLIC INVOLVEMENT (PPI)

Patient and Public Engagement has been central to the development of this research. A PPI panel was engaged through the South Central Ambulance Service Patient Forum and the Foundation Trust membership during planning and preparation of the grant application for this project. A meeting was convened where the background and ideas for the research were presented. The group could understand and articulated the overarching aim of the project. All agreed it is an important topic and the research is timely. They felt that the research was ethical and the proposed deferred consent approach was appropriate. The risks and benefits for patients of receiving study treatment were discussed with this panel. Overall, they considered that since the intervention energy levels are all used within current UK practice, recruitment into the study posed no greater risk than not being recruited into the study. I jointly drafted the lay summary of the project for the application with two members of the panel. These members wished to retain their involvement with the study and have joined the local PPI advisory panel.

Particular issues we have consulted the advisory panel on were public acceptability of the study, deferred consent and informing survivors and the relatives of non-survivors. On the issue of whether to contact the relatives of non-survivors through active or passive means, opinion was divided amongst the panel. After much discussion, it was decided that a sensitively-worded letter would be drafted with the intention of providing brief information about the study and points of contact for people to find out more if they wished. Further PPI opinion was sought on the wording

of this letter and the general approach including from members of the University Hospitals Birmingham NHS Foundation Trust Clinical Research Ambassador Group and a Patient research ambassador at the Royal Berkshire NHS Foundation Trust. This approach will be monitored throughout the study and amended if it becomes evident that the harms to relatives outweigh the benefits.

Throughout the project, two PPI members will sit on the Study Oversight Committee and we will continue to consult with, and seek the advice of, the PPI local advisory panel.

10. DISSEMINATION AND PUBLICATION

The study protocol will be published in an open-access journal. It will also be available on the university website.

The results of the study will be reported first to study collaborators. The main report will be drafted by the study co-ordinating team, and the final version will be agreed by the Study Oversight Committee before submission to the NHS Research Ethics Committee and for publication in an appropriate healthcare journal, on behalf of the collaboration. Results will be uploaded to the trials registry (ISRCTN). The success of the study depends on the collaboration of doctors, nurses and researchers from across the region. Equal credit will be given to those who have wholeheartedly collaborated in the study. Authorship of any outputs will be determined in accordance with University of Warwick SOP 22 (Publication and Dissemination) and International Committee of Medical Journal Editors guidance on authorship. The study will be reported in accordance with the Consolidated Standards of Reporting Trials (CONSORT) guidelines (www.consort-statement.org). In accordance with funder requirements, the study report will be open access.

Together with our PPI group we will construct an information sheet outlining study results. Participants, their consultees and other interested parties will be offered the opportunity to be notified of the results of the study. Those wishing to receive the results will be sent the information sheet.

Results will be shared at a national conference by members of the study team. We will also attend a local event with our PPI members and present the results of the study together.

11. REFERENCES

1. Nolan, J.P., G.D. Perkins, and J. Soar, *Improving survival after out-of-hospital cardiac arrest.* British Medical Journal, 2015.
2. Adgey, A., M. Spence, and S. Walsh, *Theory and practice of defibrillation: (2) Defibrillation for ventricular fibrillation.* Heart, 2005. **91**: p. 118-125.
3. Deakin, C. and M. Colquhoun, *Defibrillation*, in *ABC of Resuscitation*, J. Soar, G.D. Perkins, and J. Nolan, Editors. 2013, Wiley: Chichester.
4. Institute of Medicine, *Strategies to Improve Cardiac Arrest Survival: A Time to Act*, ed. R. Graham, M.A. McCoy, and A.M. Schultz. 2015, Washington, DC: The National Academies Press. 456.
5. Hawkes, C., Booth, S., Ji, C., Brace-McDonnell, S.J., Whittington, A., Mapstone, J., Cookes, M.W., Deakin, C.D., Gale, C.P., Fothergill, R., Nolan, J.P., Rees, N., Soar, J., Siriwardena, A.N., Brown, T.P., Perkins, G.D. on behalf of OHCAO collaborators, *Epidemiology and outcomes from out-of-hospital cardiac arrests in England.* Resuscitation, 2017. **110**: p. 133-140.
6. Soar, J., et al., *European Resuscitation Council Guidelines for Resuscitation 2015: Section 3. Adult advanced life support.* Resuscitation, 2015. **95**: p. 100-147.
7. Soar, J., et al., *Part 4: Advanced life support: 2015 International Consensus on Cardiopulmonary Resuscitation and Emergency Cardiovascular Care Science with Treatment Recommendations.* Resuscitation, 2015. **95**: p. e71-e120.
8. Kerber, R.D., C.D. Deakin, and W.A.J. Tacker, *Transthoracic Defibrillation*, in *Cardiac arrest: the science and practice of resuscitation medicine*, N.A. Paradis, et al., Editors. 2007, Cambridge University Press: Cambridge.
9. Morrison, L.J., et al., *Single-shock defibrillation success in adult cardiac arrest: a systematic review.* Resuscitation, 2013. **84**: p. 1480-1486.
10. Holmen, J., et al., *Survival in ventricular fibrillation with emphasis on the number of defibrillations in relation to other factors at resuscitation.* Resuscitation, 2017. **133**: p. 33-38.
11. Matsuyama, T., Kitamura, T., Kiyohara, K., Nishiyama, C., Nisiuchi, T., Hayashi, Y., Kawamura, T., Ohta, B., Iwami, T., *Impact of cardiopulmonary resuscitation duration on neurologically*

favourable outcome after out-of-hopstial cardiac arrest: A population-based study in Japan. Resuscitation, 2017. **113**: p. 1-7.
12. Moskowitz, A., et al., *In-hospital cardiac arrest: are we overlooking a key distinction?* Current opinion in critical care, 2018. **24**: p. 151-157.
13. Tomkins, W.G.O., Swain, A., Bailey, M., Larsen, P.D., *Beyond the pre-shock pause: the effect of prehospital defibrillation mode on CPR interruption and return of spontaneous circulation.* Resuscitation, 2013. **84**: p. 575-579.
14. Olsen, J.-A., Brunborg, C., Steinberg, M., Persse, D., Sterz, F., Lozano M. Jr., Westfall, M., Travis, D.T., Lerner, E.B., Brouwer, M.A., Wik, L., *Pre-shock chest compression pause effects on termination of ventricular fibrillation/tachycardia and return of organized rhythm within mechanical and manual cardiopulmoanry resuscitation.* Resuscitation, 2015. **93**: p. 158-163.
15. Stiell, I.G., Walker, R.G., Nesbitt, L.P., Chapman, F.W., Cousineau, D., Christenson, J., Bradford, P., Sookram, S., Berringer, R., Lank, P., Wells, G.A., *BIPHASIC trial: A randomized comparison of fixed lower versus escalating higher energy levels for defibrillation in out-of-hospital cardiac arrest.* Circulation, 2007. **115**: p. 1511-1517.
16. Olsen, J.-A., Brunborg, C., Steinberg, M., Persse, D., Sterz, F., Lozano M. Jr., Westfall, M., van Grunsven, P.M., Lerner, E.B., Brouwer, Wik, L., *Survival to hospital discharge with biphasic fixed 360 joules versus 200 escalating to 360 joules defibrillation strategies in out-of-hospital cardiac arrest of presumed cardiac etiology.* Resuscitation, 2019. **136**: p. 112-118.
17. Jacobs, I.G., Finn, J.C., Jeinek, G.A., Oxer, H.F., Thompson, P.L., *Effect of adrenaline on survival in out-of-hospital cardiac arrest: A randomised double-blind placebo-controlled trial.* Resuscitation, 2011. **82**: p. 1138-1143.
18. Burges Watson, D.L., Sanoff, R., Mackintosh, J.E., Saver, J.L., Ford, G.A., Price, C., Starkman, S., Eckstein, M., Conwit, R., Grace, A., Murtagh, M.J., *Evidence from the scene: paramedic perspectives on involvement in out-of-hospital research.* Annals of Emergency Medicine, 2012. **60**: p. 641-650.
19. Costa, M.L., Tutton, E., Achten, J., Grant, R., Slowther, A., *Informed consent in the context of research involving acute injuries and emergencies.* The Bone and Joint Journal, 2017. **99-B**(2): p. 147-150.
20. Davies, H., Shakur, H, Padkin, A., Roberts, I., Slowther, A.-M., Perkins, G.D., *Guide to the design and review of emergency research when it is proposed that consent and consultation be waived.* . Emergency Medicine Journal, 2014. **31**: p. 794-795.
21. Valenzuela, T.D., Roe, D.J., Nichol, G., Clark, L.L., Spaite, D.W., Hardman, B.S., *Outcomes of rapid defibrillation by security officers after cardiac arrest in casinos.* New England Journal of Medicine, 2000. **343**(17): p. 1206-1209.
22. Larsen, M., Eisenberg, M.S., Cummins, R.O., Hallstrom, A.P. , *Predicting survival from out-of-hopsital cardiac arrest: a graphic model.* Annals of Emergency Medicine, 1993. **22**(11): p. 1652-1658.
23. Perkins, G.D., Handley A.J., Koster, R.W., Castren, M., Smyth, M.A., Olasveengen, T., Monsieurs, K.G., Raffay, V., Grasner J.-T., Wenzel, V., Ristagno, G., Soar, J. on behalf of the Adult basic life support and automated external defibrillation section Collaborators *European Resuscitation Council Guidelines for Resuscitation 2015. Section 2. Adult basic life support and automated external defibrillation.* Resuscitation, 2015. **95**: p. 81-99.
24. Travers, A.H., Perkins, G.D., Berg, R.A., Castren, M., Considine, J., Escalante, R., Gazmuri, R.J., Koster, R.W.,, Lim, S.H., Nation, K.J., Olasveengen, T.M., Sakamoto, T., Sayre, M.R., Sierra, A., SMyth, M.A., Stanton, D., Vaillancourt, C., on behalf of the Basic Life Support chapter collaborators, *Part 3: Adult Basic Life Support and Automated External Defibrillation 2015 International Consensus on Cardiopulmonary Resuscitation and Emergency Cardiovascular Care Science With Treatment Recommendations.* Circulation, 2015. **132**: p. S51-S83.
25. NHS Health Research Authority, *Research in Emergency Settings*. 2017 19 Mar 2018 11 Sep 2019]; Available from: https://www.hra.nhs.uk/planning-and-improving-research/policies-standards-legislation/research-emergency-settings/.

26. Perkins, G.D., Quinn, T., Deakin, CD., Nolan, J.P., Slowther, A.-M., Cooke, M., Lamb, S.E., Petrou, S., Achana, F., Finn, J., Jacobs, I.G., Carson, A., Smyth, M., Han, K., Byers, S., Rees, N., Whitfield, R., Moore, F., Fothergill, R., Stallard, N., Long, J., Hennings, S., Horton, J., Kaye, C., Gates, S. Lall, R. , *Pre-hospital Assessment of the Role of Adrenaline: Measuring the Effectiveness of Drug administration In Cardiac arrest (PARAMEDIC-2) trial protocol.* . Resuscitation, 2016. **108**: p. 75-81.
27. Sim, J., Lewis, M., *The size of a pilot study for a clinical trial should be calculated in relation to consideration of precision and efficiency.* Journal of Clinical Epidemiology, 2012. **65**: p. 301-308.
28. Haywood, K., et al., *COSCA (Core Outcome Set for Cardiac Arrest) in Adults: An Advisory Statement From the International Liaison Committee on Resuscitation.* 2018. **137**(22): p. e783-e801.
29. Koster, R.W., R.G. Walker, and A.P. van Alem, *Definition of successful defibrillation.* Critical care medicine, 2006. **34**.
30. Ochoa, J., Ramalle-Gomara, E., Carpintero, J.M., Garcia, A., Saralegui, I., *Competence of health professionals to check the carotid pulse.* Resuscitation, 1998. **37**: p. 173-175.
31. Lapostelle, F., Le Toumelin, P., Agostinucci, J.,M., Catineau, J., Adnet, F., *Basic Cardiac Life Support Providers Checking the Carotid Pulse: Performance, Degree of Conviction, and Influencing Factors.* Academic Emergency Medicine, 2004. **11**: p. 878-880.
32. Kaji, A.H., Hanif, A.M., Thomas, J.L., Niemann, J.T., *Out-of-hospital cardiac arrest: Early in-hospital hypotension versus out-of-hospital factors in predicting in-hospital mortality among those surviving to hospital admission.* Resuscitation, 2011. **82**: p. 1314-1317.
33. Paxton, J.H., O'Neill, B.J., *When is PEA really ROSC?* Resuscitation, 2019. **142:** p.182-183.
34. Elola, A., Aramendi, E., Irusta, U., Alonso, E., Lu, Y., Chang, M.P., Owens, P., Idris, A.H., *Capnography: A support tool for the detection of return of spontaneous circulation in out-of-hospital cardiac arrest.* Resuscitation, 2019. **142:** 153- 161.
35. Bhandari, S., Doan, J., Blackwood, J., Coult, J., Kundenchuk, P., Sherman, L., Rea, T., Kwok, H., *Rhythm profiles and survival after out-of-hospital ventricular fibrillation.* . Resuscitation, 2018. **125**: p. 22-27.
36. Deakin, C.D., Kerber, R.E., *Dual sequential defibrillation: does one plus one equal two?* Resuscitation, 2016. **108**: A1-A2.
37. Perkins, G.D., et al., *Cardiac Arrest and Cardiopulmonary Resuscitation Outcome Reports: Update of the Utstein Resuscitation Registry Templates for Out-of-Hospital Cardiac Arrest.* 2015. **132**(13): p. 1286-1300.
38. Farrell, B., Godwin, J., Richards, S., Warlow, C., *The United Kingdom transient ischaemic attack (UK-TIA) aspirin trial: final results.* Journal of Neurology, Neurosurgery & Psychiatry, 1991. **54**(12): p. 1044.
39. Harrison, J.K., McArthur, K.S., Quinn, T.J. , *Assessment scales in stroke: clinimetric and clinical considerations.* Clinical Interventions in Aging, 2013. **8**: p. 201-211.
40. Nelson, M. J., Dlorio, N. M., Schmidt, T. A., Zive, D. M., Griffiths, D., & Newgard, C. D. (2013). Why persons choose to opt out of an exception from informed consent cardiac arrest trial. Resuscitation, 84, 825–830.
41. Pocock, H., Thomson, M., Taylor, S., Deakin, C.D., England, E. *Optimising ambulance service contribution to clinical trials: a phenomenological exploration using focus groups*, British Paramedic Journal, 2019. **4**(3): p.8–15.
42. O'Cathain, A., *A practical guide to using quailtative research with randomized controlled trials*. 2018, Oxford: Oxford Universtiy Press.
43. Shaw, D., & Siriwardena, A. N. (2014). *Identifying barriers and facilitators to ambulance service assessment and treatment of acute asthma: A focus group study.* BMC Emergency Medicine, 14, 18. doi: 10.1186/1471-227X-14-18.
44. Barbour, R.S., *Focus groups*, in *The Sage handbook of qualitative methods in health research.*, I. Bourgeault, Dingwall, R., De Vries, R., Editor. 2010, SAGE Publications Ltd: London. p. 327-352.

45. QSR International Pty Ltd., *NVIVO qualitative data analysis software*. 12 ed. 2018.
46. Braun, V., Clarke, V., *Using thematic analysis in psychology. Qualitative research in psychology*, 2006. **3**: p. 77-101.
47. Saver, J.L., Filip, B., Hamilton, S., Yanes, A., Craig, S., Cho, M., Conwit, R., Starkman, S., *Improving the reliability of stroke disability grading in clinical trials and clinical practice: The Rankin focussed assessment.* Stroke, 2010. **41**: p.992-995.
48. McArthur, K., Fan, Y., Pei, Z., Quinn, T., *Optimising outcomes assesment to improve quality and efficiency of stroke trials,* Expert Reviews in Pharmacoeconomic Outcomes Research, 2014. **14**(1): p.101-111.
49. Health Research Authority, M.H.R.A., Devolved administrations of Northern Ireland, Scotland & Wales, *Joint statement on the application of Good Clinical Practice to training for researchers*. 2017.
